# Predicting and Understanding the Pathology of Single Nucleotide Variants in Human *COQ* Genes

**DOI:** 10.3390/antiox11122308

**Published:** 2022-11-22

**Authors:** Sining Wang, Akash Jain, Noelle Alexa Novales, Audrey N. Nashner, Fiona Tran, Catherine F. Clarke

**Affiliations:** Department of Chemistry & Biochemistry, The Molecular Biology Institute, UCLA, Los Angeles, CA 90095, USA

**Keywords:** coenzyme Q, ubiquinone, primary CoQ deficiency, mitochondrial disease, single nucleotide variants, Missense3D, *COQ* genes

## Abstract

Coenzyme Q (CoQ) is a vital lipid that functions as an electron carrier in the mitochondrial electron transport chain and as a membrane-soluble antioxidant. Deficiencies in CoQ lead to metabolic diseases with a wide range of clinical manifestations. There are currently few treatments that can slow or stop disease progression. Primary CoQ_10_ deficiency can arise from mutations in any of the *COQ* genes responsible for CoQ biosynthesis. While many mutations in these genes have been identified, the clinical significance of most of them remains unclear. Here we analyzed the structural and functional impact of 429 human missense single nucleotide variants (SNVs) that give rise to amino acid substitutions in the conserved and functional regions of human genes encoding a high molecular weight complex known as the CoQ synthome (or Complex Q), consisting of the *COQ3*–*COQ7* and *COQ9* gene products. Using structures of COQ polypeptides, close homologs, and AlphaFold models, we identified 115 SNVs that are potentially pathogenic. Further biochemical characterizations in model organisms such as *Saccharomyces cerevisiae* are required to validate the pathogenicity of the identified SNVs. Collectively, our results will provide a resource for clinicians during patient diagnosis and guide therapeutic efforts toward combating primary CoQ_10_ deficiency.

## 1. Introduction

Coenzyme Q (CoQ), also known as ubiquinone, is a redox-active lipophilic molecule required for cellular respiration. Its structure consists of a tetra-substituted benzoquinone ring and includes a lipid-anchoring polyisoprenyl tail of variable unit-length (CoQn, *n* = ten isoprene units in humans, CoQ_10_) [1]. In the mitochondria, CoQ functions as an electron carrier in metabolic processes, such as oxidative phosphorylation, fatty acid β-oxidation, and choline metabolism [1,2]. In its oxidized form, CoQ accepts electrons from Complex I, Complex II, and many other mitochondrial dehydrogenases, producing its reduced form, ubiquinol (CoQH_2_), which donates electrons and protons to Complex III [1,2,3]. In addition, CoQH_2_ serves as a general lipophilic antioxidant [4,5,6] that has been implicated in protection from lipid autoxidation [7,8], and regeneration of vitamin E [9], another small molecule antioxidant. More recently, CoQH_2_ has also been identified as having a role in ferroptosis suppression [10,11].

**Figure 1:**
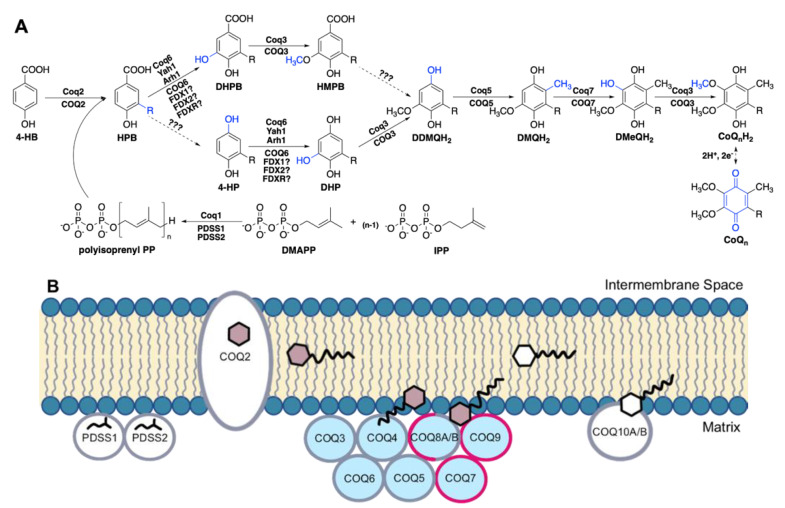
Biosynthesis of coenzyme Q. (**A**) The CoQ biosynthetic pathway is largely homologous between *S. cerevisiae* (polypeptide names above arrows) and humans (polypeptide names below arrows). In humans, at least seven nuclear-encoded catalytic proteins are directly responsible for the biosynthesis of CoQ from 4-hydroxybenzoic acid (4-HB), a tyrosine derivative. The enzyme(s) responsible for the decarboxylation and hydroxylation step(s) (dashed arrows) at ring position 1 has not been found. Hence, there is uncertainty about the order of steps. The decarboxylation step may precede the Coq6/COQ6 hydroxylation step based on the accumulation of 4-hydroxy-3-polyprenylphenol (4-HP) in yeast and human cells harboring mutations in Coq6/COQ6 data from [14,33,34]. In addition to the yeast Coq and human COQ polypeptides, other polypeptides involved in CoQ biosynthesis include PDSS1 and PDSS2 (decaprenyl diphosphate synthase subunits 1 and 2), Yah1 (yeast ferredoxin), Arh1 (yeast ferredoxin reductase), FDX1 and FDX2 (human ferredoxins 1 and 2), and FDXR, human ferredoxin reductase. Intermediates in the pathway include: DMAPP, dimethylallyl pyrophosphate; IPP, isopentenyl pyrophosphate; HPB, 3-polyprenyl-4-hydroxybenzoic acid; DHPB, 4,5-dihydroxy-3-polyprenylbenzoic acid; HMPB, 4-hydroxy-5-methoxy-3-polyprenylbenzoic acid; DHP, 4,5-dihydroxy-3-polyprenylphenol; DDMQH_2_, 2-methoxy-6-polyprenyl-1,4-benzohydroquinone; DMQH_2_, 2-methoxy-5-methyl-6-polyprenyl-1,4-benzohydroquinone; DMeQH_2_, 3-methyl-6-methoxy-2-polyprenyl-1,4,5-benzenetriol. Note that the intermediates found in *S. cerevisiae* contain a hexaprenyl tail, while humans make decaprenylated CoQ_10_ intermediates. (**B**) The CoQ synthome (Complex Q in humans) is a high-molecular mass protein and lipid complex data from [1], consisting of polypeptides COQ3-COQ9. The PDSS proteins (homologous to *S. cerevisiae* Coq1), COQ2, and the lipid-binding proteins COQ10A and COQ10B do not associate with the complex. Colored hexagons indicate CoQ intermediates; white hexagons indicate the final CoQ product. Polypeptides for which the human protein structures have been solved are highlighted in pink. The COQ8A/COQ8B polypeptides are thought to have a dynamic association with complex Q. Polypeptides are not drawn to scale and their stoichiometry has not been determined.

In humans, the biosynthesis of CoQ begins with 4-hydroxybenzoic acid (4-HB), a tyrosine derivative [12] (Figure 1A). The bulk of CoQ biosynthesis occurs in the mitochondrial matrix, where at least thirteen nuclear-encoded COQ polypeptides (PDSS1, PDSS2, COQ2-COQ7, COQ8A (ADCK3), COQ8B (ADCK4), COQ9, COQ10A, and COQ10B) reside in or are peripherally associated with the inner mitochondrial membrane (IMM) [13] (Figure 1B). In addition, ferredoxin Yah1 (human homolog FDX1 and FDX2) and ferredoxin reductase Arh1 (human homolog FDXR) from *Saccharomyces cerevisiae* have been shown to be important for CoQ biosynthesis through the monooxygenation step performed by Coq6, although the involvement of their human homologs in CoQ biosynthesis has not been demonstrated [14]. The PDSS1 and PDSS2 polypeptides catalyze the formation of the decaprenyl diphosphate tail characteristic of CoQ_10_, and COQ2 attaches the tail to 4-HB [15]. The PDSS1/PDSS2 complex and the COQ2 polypeptides appear to work independently. Polypeptides COQ3–COQ7 and COQ9 assemble into a high molecular weight complex, termed the CoQ synthome (or complex Q), which has been confirmed by mass spectrometry [16] and co-purification methods [17,18,19,20]. COQ8A and COQ8B are reported to have dynamic interactions with the remaining protein components [16]. Similarly in yeast, while the Coq8 polypeptide is necessary for CoQ synthome assembly [21,22] and co-purified with the Coq6 polypeptide [23], fluorescence microscopy of yEGFP-tagged Coq8 reveals the protein is dispersed throughout the mitochondria, forming few domain-like patterns, unlike the remaining polypeptides in the core complex that specifically reside in discrete puncta [24]. In addition to protein components, this CoQ synthome in yeast and complex Q in human cells also contains lipid intermediates and small molecule ligands [23,24,25,26,27].

Mutations in the genes encoding COQ polypeptides lead to primary CoQ_10_ deficiency, as they directly affect the biosynthesis of CoQ. This rare condition is typically caused by autosomal recessive mutations and often results in highly variable clinical manifestations, ranging from isolated pathologies, such as in the kidneys or central nervous system (CNS), to a fatal multi-system disorder [28,29,30]. The high variability may be attributed to discrepancies in genetic backgrounds, variable tissue-specific expression levels, and a varying degree of pathogenicity for each mutation. Systems and tissues generally impacted by primary CoQ_10_ deficiencies include the CNS, heart, peripheral nervous system (PNS), kidneys, and muscles. Some rarer manifestations can involve the lungs, liver, thyroid, and more general metabolic or cardiovascular disorders [28,29]. To date, there are approximately 200 patients with primary CoQ_10_ deficiency described in the literature, spanning mutations in ten of fourteen genes encoding COQ polypeptides [28,29]. Additionally, secondary CoQ_10_ deficiencies can result from aging or treatment with statins and have also been linked to mutations in genes such as *APTX* (aprataxin), *BRAF* (B-Raf), *ETFDH* (mitochondrial electron transfer flavoprotein-ubiquinone oxidoreductase), and *MUT* (methylmalonyl-CoA mutase), that are not directly linked to the biosynthesis of CoQ_10_ [1]. Currently, the only treatment for CoQ_10_ deficiency is exogenous CoQ_10_ supplementation. However, therapeutic benefits of exogenous supplementation are limited due to the extreme hydrophobicity and low bioavailability of CoQ_10_ [31]. Supplementation with CoQ_10_ may slow or stop the progression of disease but cannot reverse the damage already incurred in the patient [30]. In cases of early intervention in patients diagnosed with CoQ_10_ deficiency-associated nephropathy, CoQ_10_ supplementation has been shown to be remarkably effective in resolving the nephrotic syndrome, underscoring the significance of prompt intervention [32].

In this study, we utilized exome and genome sequencing data of large populations, as well as clinical variants reported in the literature and the NCBI ClinVar database [35] to generate a comprehensive list of missense single nucleotide variants (SNVs) found in genes encoding the core CoQ synthome or complex Q (*COQ3*–*COQ7* and *COQ9*). The *COQ8A* and *COQ8B* genes were excluded due to their aforementioned dynamic interactions with the core complex. We structurally and functionally characterized variants that occurred in conserved and functional regions of the protein, using multiple sequence alignment and structural analyses with available models and crystal structures. In addition, clinically reported variants and those with high allele frequency were also included in our analyses. Our final list consisted of 429 variants compiled from gnomAD [36], ClinVar, Missense3D-DB [37], as well as the published research literature. We predicted the pathogenicity of each variant using the Missense3D mutation classifier, which relies purely on structural information and local structural changes [38]. When an experimentally determined structure was not available, as is often the case for the COQ polypeptides, Missense3D presented the advantage of being able to make predictions based on computational models such as those generated by AlphaFold [39]. This is in contrast to other classifier methods, including SIFT [40] and Polyphen-2 [41], that rely on evolutionary conservation and a mixture of sequence- and structure-based predictive features, respectively. In total, Missense3D identified 115 variants that are structurally damaging and potentially pathogenic. Confirmation of these predictions will require biochemical characterization in model systems, such as *Saccharomyces cerevisiae*. The results presented will provide a resource for clinicians with the aim of guiding efforts toward the treatment of primary CoQ_10_ deficiency.

## 2. Materials and Methods

### 2.1. Multiple Sequence Alignment

All human COQ polypeptide and orthologous sequences were obtained from NCBI GenBank Release 246.0 [42]. Multiple sequence alignments were generated using the ClustalW package of Clustal Omega [43] and visualized with Jalview2 [44]. Residues were classified as highly conserved if the percent agreement at that position was higher than 80%, as calculated by the built-in feature of Jalview and indicated by a dark blue shading in the sequence alignment. Prior biochemical characterization and crystal structure of *Escherichia coli* ubiquinone biosynthesis *O*-methyltransferase UbiG (the COQ3 ortholog) was used to determine the functional regions in human COQ3 (PDB: 4KDC and 5DPM) [45,46]. Molecular genetic analyses of *S. cerevisiae* Coq4 were used to determine the potential zinc-liganding residues in human COQ4 [47]. Apoenzyme (apo; PDB: 4OBX) and AdoMet-bound (PDB: 4OBW) form of *S. cerevisiae* Coq5 crystal structures were used to determine homologous residue functionality in human COQ5 [48]. Functional residues in human COQ6 were determined using an *S. cerevisiae* Coq6 model [49]. Prior biochemical characterization, a structural model, and a recent structure of human COQ7 were used to identify the functional regions of COQ7 [50,51,52]. Prior biochemical characterization, crystal structures, and molecular dynamics simulations were used to outline the functional residues in COQ9 (PDB: 6AWL and 6DEW) [50,52].

### 2.2. Analysis of Single Nucleotide Variants

Human SNVs occurring in the canonical transcripts, as reported in NCBI RefSeq [53] for *COQ3*–*COQ7* and *COQ9*, were gathered from gnomAD v2.1.1 [36], NCBI ClinVar [35], Missense3D-DB v1.5.1 [37], and available literature. Only SNVs that resulted in a missense variant were analyzed. In addition, a filter was applied to retain variants that occur in highly conserved (see Section 2.1) or functional amino acid positions. Variants documented in the ClinVar database or literature, as well as variants with an allele frequency larger than 1.00 × 10^−4^ as reported in gnomAD v2.1.1 (labeled as “Frequent Polymorphisms”), were also included in our analysis. Finally, exonic variants that are within two nucleotides of a splice site were included as variants of potential interest. Since the Missense3D-DB had already published variant classification using the crystal structure of COQ9, any variant that was reported as structurally damaging in the database was also included in our analysis, regardless of amino acid position or conservation.

**Figure 2:**
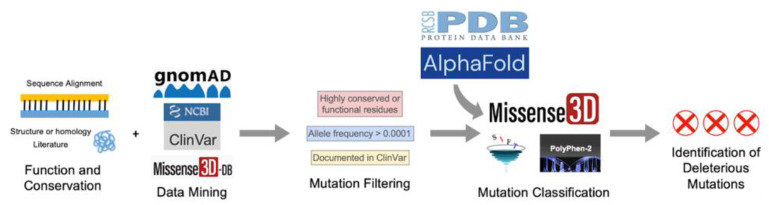
Schematic diagram of the overall approach used in this study.

The selected variants were classified with three mutation classifiers, each using a different algorithm. Missense3D (http://missense3d.bc.ic.ac.uk/missense3d/, accessed on 30 March 2022) reports deleteriousness based on local structural feature changes that may be destabilizing [38]. SIFT (https://sift.bii.a-star.edu.sg/, accessed on 30 March 2022) scores variants as “Tolerated” or “Deleterious” based on evolutionary conservation [40]. Polyphen-2 (http://genetics.bwh.harvard.edu/pph2/, accessed on 30 March 2022) uses a Naïve Bayes classifier to report the likelihood of a mutation being damaging based on a combination of sequence- and structure-based criteria, whenever structures are available. Variants are scored as “Benign”, “Possibly Damaging”, and “Probably Damaging” [41]. Default settings were used whenever applicable. Results and interpretations from the three mutation classifiers can be found in Supplemental Table S1. The pipeline for SNV selection and classification is illustrated in Figure 2.

### 2.3. Missense3D Analysis

Selected variants were submitted to the Missense3D online server for analysis of structural impact. For COQ3–COQ7, all variants were analyzed using the structural predictions of the human protein obtained from the AlphaFold Protein Structure Database [39,54]. For COQ9, variants occurring in regions modeled by its crystal structure (PDB 6AWL) were assessed using the crystal structure, while the remaining variants were assessed using their AlphaFold models. AlphaFold models used in this study include Q9NZJ6 (COQ3), Q9Y3A0 (COQ4), Q5HYK3 (COQ5), Q9Y2Z9 (COQ6), Q99807 (COQ7), and O75208 (COQ9). Structures were visualized using PyMOL (PyMOL Molecular Graphics System, Version 2.4.2, Schrödinger, LLC, New York, NY, USA).

## 3. Results

### 3.1. COQ3

COQ3 is an *S*-adenosyl-L-methionine (AdoMet)-dependent methyltransferase that catalyzes both *O*-methylation steps in CoQ biosynthesis in both *S. cerevisiae* and humans [55,56,57]. Namely, it is responsible for the conversion of DHPB to HMPB as well as the last step in CoQ biosynthesis, DMeQH_2_ to CoQH_2_ (Figure 1A). Overexpression of human COQ3 has been shown to rescue the growth CoQ biosynthesis of respiratory deficient *coq3Δ* yeast [56]. COQ3 and its yeast homolog are required for synthome stabilization, as partial knockdown of COQ3 in human cells results in a significant decrease in the levels of COQ4–COQ9, with the exception of COQ5 [58]. This result is corroborated by studies in yeast harboring a deletion in *COQ3* [59]. Interestingly, Coq3 is stable in *coq4–coq9* null mutant yeast strains studied in the presence of phosphatase and protease inhibitors, and its level is dependent on Coq8 [21,59]. There are currently no crystal structures available for eukaryotic homologs of COQ3. Structures are available for the orthologous *O*-methyltransferase UbiG from *E. coli*, which shares 34% sequence identity with human COQ3 and is part of a soluble ubiquinone/menaquinone biosynthetic complex [60]. Crystal structures of the apo and S-adenosyl-L-homocysteine (AdoHcy)-bound UbiG monomer have guided subsequent biochemical characterizations and confirmed its seven β-strand Rossmann-fold structure [45,46].

To date, there have not been reported cases of primary CoQ_10_ deficiency associated with mutations in the *COQ3* gene [28,29,30], and pathogenic SNVs have not been identified. The lack of clinical data is echoed in yeast studies, which have primarily focused on *COQ3* gene deletions. As a result, no point mutations conferring pathogenic effects have been characterized in yeast. However, *COQ3* gene expression was found to be upregulated in esophageal squamous cell carcinoma and was associated with poor prognosis, hinting at a potentially unique role of COQ3 in humans [61,62].

**Figure 3:**
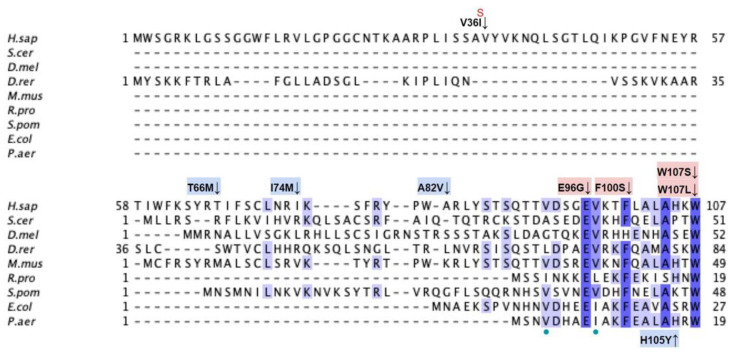

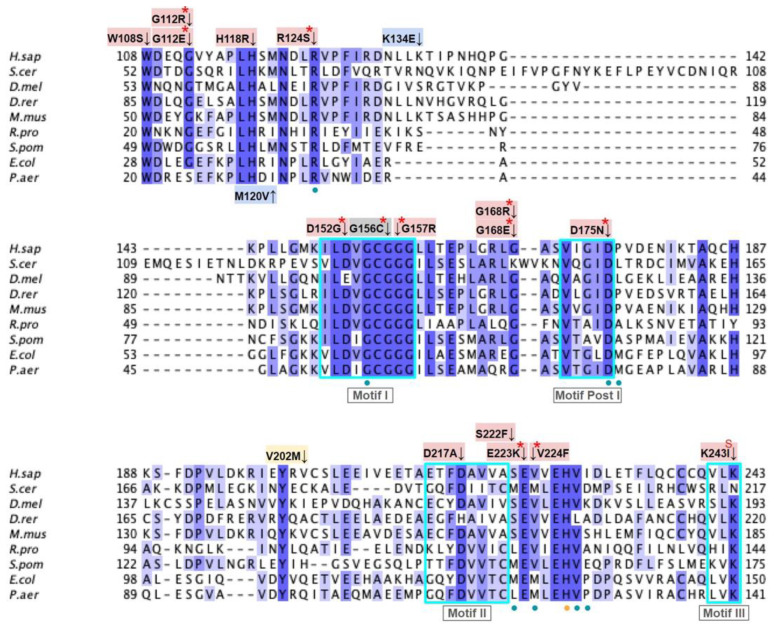

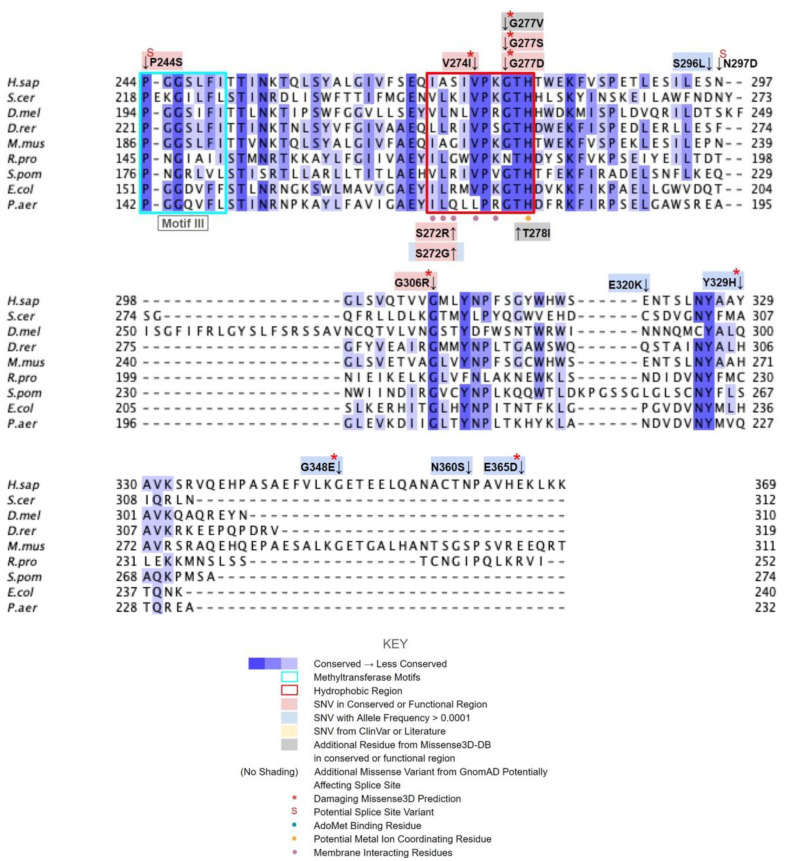
Labeled and annotated multiple sequence alignment of COQ3. Amino acid sequences of COQ3 were analyzed as described in Materials and Methods and include *Homo sapiens* (NCBI accession number NP_059117.3) and homologs in *Saccharomyces cerevisiae* (NP_014545), *Drosophila melanogaster* (NP_610092.2), *Danio rerio* (NP_001002620.1), *Mus musculus* (NP_766275.1), *Rickettsia prowazekii* (WP_004596275.1), *Schizosaccharomyces pombe* (NP_588239.2), *Escherichia coli* (NP_416735.1), and *Pseudomonas aeruginosa* (WP_003122245.1). See KEY for descriptions of the figure annotations. Putative AdoMet binding residues are denoted by a blue dot, and residues thought to interact with the cell membrane in *E. coli* UbiG are denoted by a purple dot, data from [45,46]. A pair of co-evolving, highly conserved, and structurally nearby histidines may be involved in metal ion coordination (orange dot), data from [63]. Methyltransferase motifs I, post-I, II, and III are boxed in cyan data from [56,64]. The putative membrane-interacting hydrophobic region is boxed in red, data from [45]. For all multiple sequence alignments, conservation is indicated via shaded residues, which represent a percent identity great than 80%, 60%, and 40%, from darkest to lightest. Residues with less than 40% identity are unshaded.

From gnomAD, ClinVar, and Missense3D-DB, we identified 44 missense SNVs of interest (Figure 3; Supplemental Table S1; see Section 2.2 for the selection criteria). Of these variants, 29 coincided with a highly conserved region or functional residue determined from structural and biochemical studies, as well as analyses of sequence conservation [45,46,63]. Sixteen SNVs occurring in highly conserved or functional residues were classified as structurally damaging by Missesense3D. Twelve additional variants from gnomAD that were neither highly conserved nor functional residues were included due to their notable allele frequency. Interestingly, three of these frequent variants were also classified as structurally damaging. V202M was the only variant listed in ClinVar, although its clinical significance was “Likely Benign”. Finally, a total of four variants were marked as potential splice site variants in addition to their apparent amino acid sequence change. Since the effect on splicing may be confounding, this group of variants was not subjected to extensive study.

**Figure 4:**
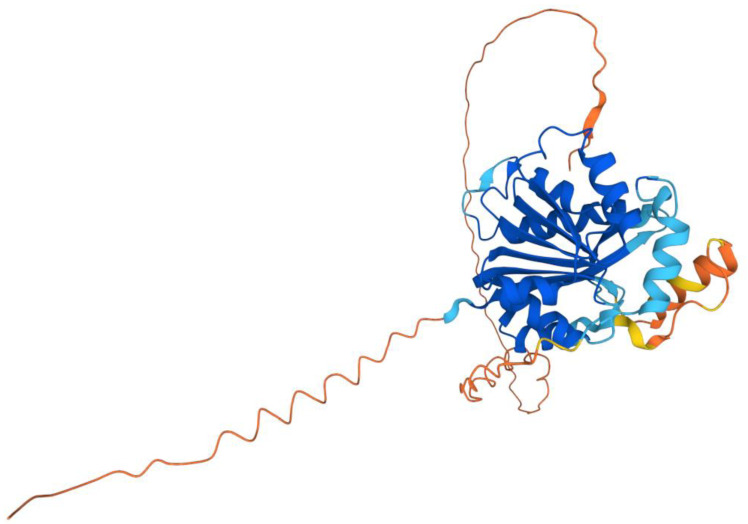
AlphaFold model of human COQ3. Model color corresponds to model confidence in each region. Very high confidence in dark blue (pLDDT > 90); confident in light blue (90 > pLDDT > 70); low confidence in yellow (70 > pLDDT > 50); very low confidence in orange (pLDDT < 50). pLDDT is a measure of per-residue confidence. pLDDT < 50 is a reasonably strong predictor of disorder for the corresponding residue. Figure was generated by the AlphaFold Protein Structure Database; adapted with permission from [39,54].

**Figure 5:**
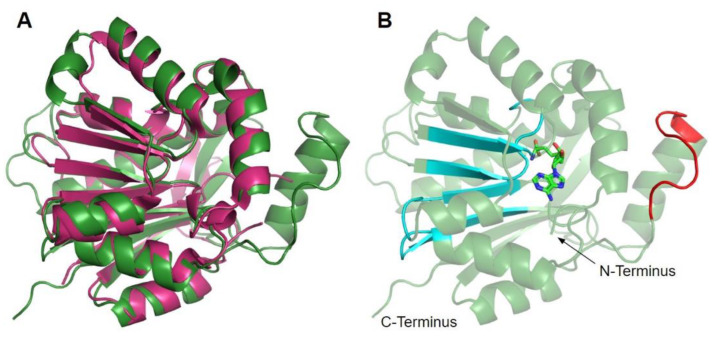
Alignment of AlphaFold-generated model of human COQ3 with existing *E. coli* UbiG crystal structure. (**A**) Superimposition of the human COQ3 model (residues 91–336, shown in dark green), adapted with permission from [39,54], and the crystal structure of AdoHcy-bound *E. coli* UbiG (shown in magenta, PDB: 5DPM). The N- and C-termini of the model were omitted due to low confidence. Note that this and subsequent figures about COQ3 were generated using PyMOL, which classifies a short-coil region as a helix (top left of figure). This gives a total of nine helices as opposed to eight rendered in Figure 4. (**B**) Human COQ3 model with conserved methyltransferase motifs I–III and post-I highlighted in cyan and the hydrophobic region in red. AdoMet (shown in light green) was modeled via structural alignment with PDB 5DPM using PyMOL.

**Figure A1:**
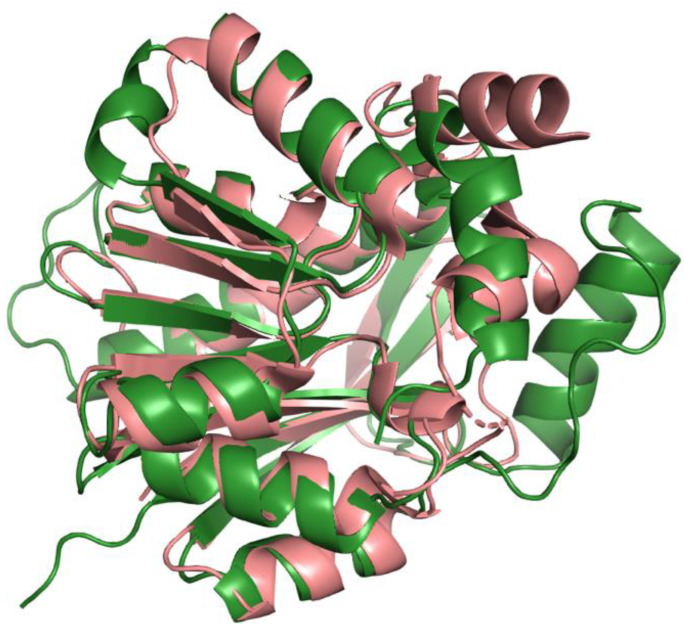
Structural alignment of the human COQ3 model (shown in dark green) and the crystal structure of *E. coli* UbiG, apo form (shown in pink, PDB: 4KDC). Residues 1–90 and 337–369 of the AlphaFold model were omitted due to disordered structure. Alignment was performed using PyMOL.

The human COQ3 model was obtained from the AlphaFold Protein Structure Database (Figure 4) [39,54]. The core structure of the model consists of eight α-helices (as rendered by the AlphaFold 3D viewer) and eight β-sheets, flanked by long disordered regions on the N- and C-termini. The overall structure of the model aligned well with the crystal structure of AdoHcy-bound (PDB: 5DPM; Figure 5A) and apo *E. coli* UbiG (PDB: 4KDC; Figure A1). Four sequence motifs shared among class I methyltransferases, motifs I–III and post-I, each form a β-strand that aligns the residues in the AdoMet binding pocket (Figure 5B) [64,65]. In addition to the conserved Rossmann fold structure, the UbiG/COQ3 family contains a hydrophobic stretch of ten residues (residues 270–279 in the human COQ3 sequence) that is thought to interact with the membrane in UbiG [46]. An isolated helix linked to the hydrophobic region is visible in the COQ3 model, while this region was truncated in the crystal structure of UbiG (Figure 5A) [46]. This region is thought to act as a gate to the AdoMet binding pocket, which is consistent with its position in the model (Figure 5B) [46]. However, it is not clear how this putative membrane interaction helix is compatible with the recent structure of the soluble Ubi metabolon [66].

#### 3.1.1. SNVs in Methyltransferase Motifs I and Post I

**Figure 6:**
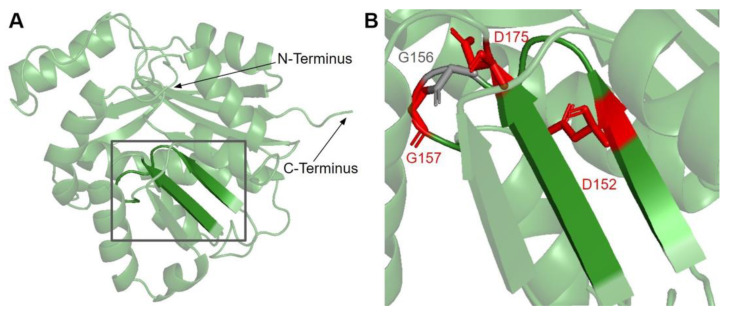
SNVs found in motifs I and post I of COQ3. (**A**) Motifs I (right β-strand and loop, residues I150 to G1158) and post I (left β-strand, residues V171 to D175) highlighted on residues 91–336 of the COQ3 model. N- and C-termini were truncated for simplicity. (**B**) Locations of SNVs in motifs I and post-I are depicted. Residues are colored according to their corresponding SNVs in Figure 3.

Motif I (residues I150 to G1158) is found in the first β-strand followed by a Gly-rich loop that extends into the putative AdoMet binding site of COQ3 (Figure 6A) [65]. It closely resembles the nine-residue consensus sequence, with two aliphatic residues followed by a (D/E) (V/I) GXGXG motif [64]. The backbone carbonyl of the G66 in *E. coli* in this motif (G156 in humans) has been shown to form a hydrogen bond with the amino group of AdoHcy in UbiG (PDB: 5DPM) [46].

There are three SNVs of interest in this region, all of which occur on highly conserved residues (Figure 3 and Figure 6B). D152G from gnomAD maps to the β-strand region, which is critical for the alignment of AdoMet binding residues. Missense3D classifies this variant as structurally damaging due to the replacement of a buried charge and the loss of a buried hydrogen bond. These disruptions may lead to destabilization of the core structure and more profound changes to the CoQ synthome. G156C and G157R were reported in Missense3D-DB and gnomAD, respectively. These two SNVs map to the flexible Gly-rich loop. Missense3D classifies both variants as structurally damaging due to the replacement of buried Gly residues. In addition, G156C was flagged for altering the buried/exposed status of this residue position, which likely refers to the mutant Cys residue protruding into the AdoMet binding site. G157R, on the other hand, introduces a buried charge in proximity with the putative docking region of the amino and carboxyl groups of AdoMet. Indeed, mutating the homologous residue in yeast, G133, to an Ala resulted in partial suppression of respiratory growth and significantly reduced CoQ levels, despite stable expression of the polypeptide [63]. Taken together, these two variants likely affect cofactor binding and enzyme activity.

Motif post-I (V171 to D175) is part of the second β-strand that contains a short stretch of hydrophobic residues followed by a highly conserved acidic residue (Figure 6A) [65]. The acidic residue in *E. coli* UbiG, D85 (D175 in humans) was shown to form hydrogen bonds with the O2′ and O3′ hydroxyl groups on the ribose ring of AdoHcy in the crystal structure of UbiG (PDB: 5DPM), while residue M86 (P176 in humans) immediately following motif post-I interacts with the adenine ring [46]. D175N is the only SNV of interest in this region (Figure 3 and Figure 6B). This variant was classified as structurally damaging by Missense3D due to the replacement of a buried charge, which may disrupt the aforementioned interactions with the ribose ring.

#### 3.1.2. SNVs in Methyltransferase Motifs II and III

**Figure 7:**
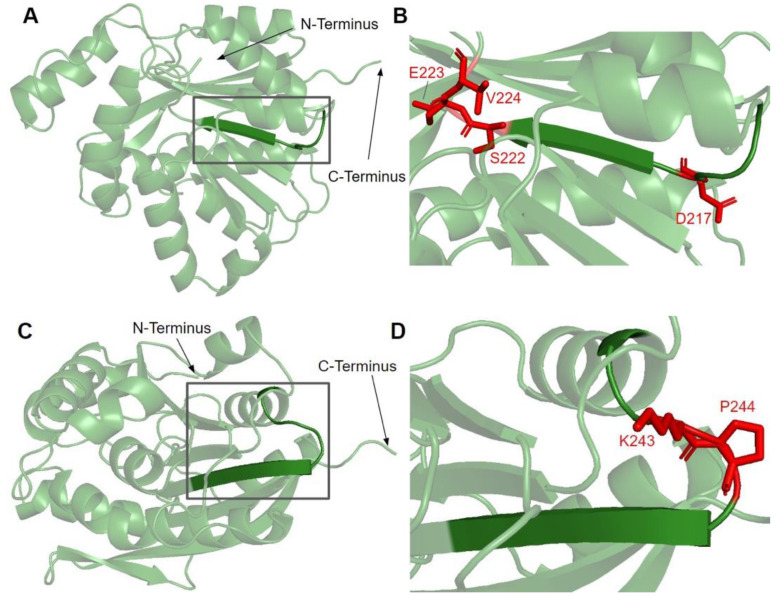
SNVs found in motifs II and III of COQ3. (**A**,**C**) Residues 91–336 of the COQ3 model with E214 to A221 as motif II (**A**) and V241 to I250 as motif III (**C**) are highlighted. (**B**,**D**) Locations of SNVs in motif II and nearby residues (**B**) and motif III (**D**) are depicted. N- and C-termini were truncated for simplicity. Residues are colored according to their corresponding SNVs in Figure 3.

Motif II, roughly E214 to A221, maps to residues immediately preceding and including the fourth β-strand (Figure 7A). The sequence of motif II is not as well conserved among COQ3 homologs, although one highly conserved Asp is thought to make contact with motif I (Figure 3) [65]. Aliphatic residues following this Asp mark the start of β4, which leads to a loop containing residues forming a hydrophobic environment for the adenine ring of AdoHcy in UbiG [46]. In addition, this region following motif II is thought to interact with the methyl group in AdoMet as well as the substrate, giving it a possible role in determining substrate specificity [65,67]. This hypothesis is supported by coevolution studies of UbiG/COQ3, in which residue position 196 in yeast Coq3 (222 in humans) was shown to coevolve in other methyltransferases but was distinct in Coq3 and *E. coli* UbiG. One additional residue of interest downstream is H227 in human COQ3. Its homologous residue in UbiG, H134, was shown to be required for growth in *E. coli*, and in the *Arabidopsis thaliana* small RNA 2′-O-methyltransferase HEN1 the homologous residue was described as magnesium-binding [45,68]. Mutating this residue to an Ala in yeast (H201A) completely destabilized the polypeptide [63]. In motif II and the loop following it, there are four SNVs of interest in highly conserved or functional residues, all of which were reported in gnomAD (Figure 3 and Figure 7B). D217A immediately preceding β4 was, while highly conserved, not classified as damaging by Missense3D due to the residue being solvent-exposed in the model. S222F, E223K, and V224F are located in the loop following β4. S222 and V224 are thought to bind the amino group and the adenine ring of AdoMet, respectively, by homology with *E. coli* UbiG (M129 and M131) [46]. However, only V224F was classified as structurally damaging due to a change in cavity size, likely referring to the binding pocket for the adenine ring of AdoMet. S222F and E223K were also solvent-exposed in the model, which resulted in their classification as neutral despite their disruption of hydrogen bonds or salt bridges. In yeast, an Ala mutation in the homologous position of E223 completely destabilized the Coq3 polypeptide and disrupted respiratory growth. Coq4 was also destabilized as a consequence [63]. This strongly suggests that all three variants in the loop are likely disruptive.

Motif III (V241 to I250) lies on the fifth β-strand as well as the loop and parts of the helix preceding it (Figure 7C). This loop is essential for the alignment of the methyltransferase motifs and consists of Pro and Gly residues to facilitate the turn [65]. GnomAD reported two SNVs, K243I and P244S, in highly conserved residues of this motif (Figure 7D). Neither SNV was classified as structurally damaging by Missense3D. However, it is worth noting that these SNVs occur near a splice junction and may therefore result in more profound changes to protein expression in addition to changes in protein sequence. 

#### 3.1.3. SNVs in the Membrane Interacting Hydrophobic Region

**Figure 8:**
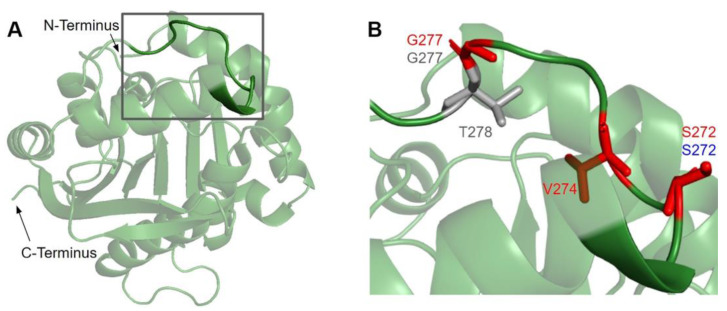
SNVs found in the membrane interacting hydrophobic region of COQ3. (**A**) Residues I270 to H279 are highlighted on residues 91–336 of the COQ3 model. N- and C-termini were truncated for simplicity. (**B**) Locations of SNVs in the hydrophobic region are depicted. Residues are colored according to their corresponding SNVs in Figure 3.

Previous studies demonstrated that a region consisting of several hydrophobic and basic residues in *E. coli* UbiG and human COQ3 is required for their interaction with liposomes containing cardiolipin [45]. Furthermore, mutating multiple residues in this region to Ala resulted in reduced growth in *E. coli* [45]. In the human COQ3 model, this region can be seen mapping to a linker connecting an isolated α-helix to the core structure (Figure 8A). This is consistent with a model in UbiG in which this region acts as a gate to the binding site of the AdoMet cofactor [46]. Upon binding to the membrane, this helix is thought to dissociate from the core structure, allowing the cofactor to diffuse into the active site. Mutating residues in and preceding this region to disrupt its interaction with the core structure indeed resulted in enhanced binding of AdoHcy [46]. Nevertheless, it is worth noting that there are key differences in CoQ biosynthesis in *E. coli* and humans. UbiG is part of a soluble complex in the cytosol, while COQ3 is part of a membrane-associated complex in the mitochondrial matrix [60]. Implications of this membrane interaction in human COQ3 have not been investigated. Its preferential binding to liposomes containing cardiolipin, a unique component of the inner mitochondrial membrane, suggests a similar cofactor gating role in human COQ3. Meanwhile, a more recent coevolution study of the UbiG/COQ3 family identified the His residue at the end of this region (H279 in humans, H254 in yeast) as coevolving with the putative metal binding H201 in yeast (H227 in humans) [63]. The close proximity of the two His residues in our model supports an alternative role of metal binding. However, the functional significance of this region in human COQ3 remains unelucidated.

In this region, we have identified seven variants spanning five residue positions from gnomAD and Missense3D-DB (Figure 8B). S272G, S272R, and V274I all coincide with residues known to affect liposome binding in UbiG when mutated [45]. Interestingly, S272G is a frequent polymorphism with an allele frequency of 8.45 × 10^−1^, suggesting that this variant may be benign. V274I was classified as structurally damaging by Missense3D due to changes in cavity size, which likely refers to the active site of COQ3. This classification suggests a possible change in either substrate or cofactor binding and therefore activity. G227S, G227V, G227D, and T278I were not tested in UbiG, although the high conservation suggests functional importance. All three variants occurring on G227 were classified as damaging by Missense3D due to disallowed phi/psi angle.

#### 3.1.4. Frequent Polymorphisms in COQ3

In this study, we have defined “frequent polymorphism” as a variant having an allele frequency larger than 1.00 × 10^−4^. There are 12 such variants found in COQ3 that do not fall into the previously described functional regions. Most of them occur in regions of poor conservation such as the N- and C-termini. Two variants, Y329H and K134E, stand out with their exceptionally high allele frequency of 9.84 × 10^−1^ and 3.03 × 10^−1^, respectively. Interestingly, Y329H, located on β7, was classified as structurally damaging by Missense3D due to the introduction of a buried charge. However, given the large number of homozygotes that result from its allele frequency, this variant is unlikely to be detrimental. Two additional variants, G348E (allele frequency 8.14 × 10^−4^) and E365D (allele frequency 6.07 × 10^−4^), were classified as structurally damaging. The former was flagged for replacement of a Gly residue in a bend, which may affect the flexibility of the region. The latter was flagged for steric clash, which may be an artifact of the Missense3D program since the region is disordered. 

### 3.2. COQ4

COQ4 has not been characterized as having an enzymatic function, though it appears to play an important structural role in organizing the CoQ synthome [13]. Human transcripts encode two *COQ4* isoforms; isoform 2 lacks both the first exon and the mitochondrial leader sequence and fails to rescue yeast *coq4* null mutants [69]. The polypeptide encoded by *COQ4* isoform 1 complements *coq4* null mutant yeast [69]. This demonstrates conserved functionality across species as well as the power of *S. cerevisiae* as a model organism to study human *COQ4* mutations [69]. Human COQ4 is located within mitochondria [69], as is the yeast Coq4 polypeptide that is peripherally associated with the matrix side of the IMM [70].

The first structure determined for a COQ4 homolog, the Alr8543 polypeptide from *Nostoc* sp. (PDB: 3KB4), revealed a geranylgeranyl monophosphate bound between hydrophobic helices. However, PDB: 3KB4 is now obsolete and has been replaced with PDB: 6E12. The Alr8543 protein in the more recent 6E12 structure is in complex with two magnesium ions. Both Mg^2+^ ions are associated with a conserved HDXXH-(X)_10–13_-E signature motif required for COQ4 functionality and is similarly considered to chelate a zinc ion [47]. Mutations in the human *COQ4* gene are associated with primary CoQ_10_ deficiency and with several disease states including a range of neurological afflictions, cardiomyopathy, and respiratory distress [29].

**Figure 9:**
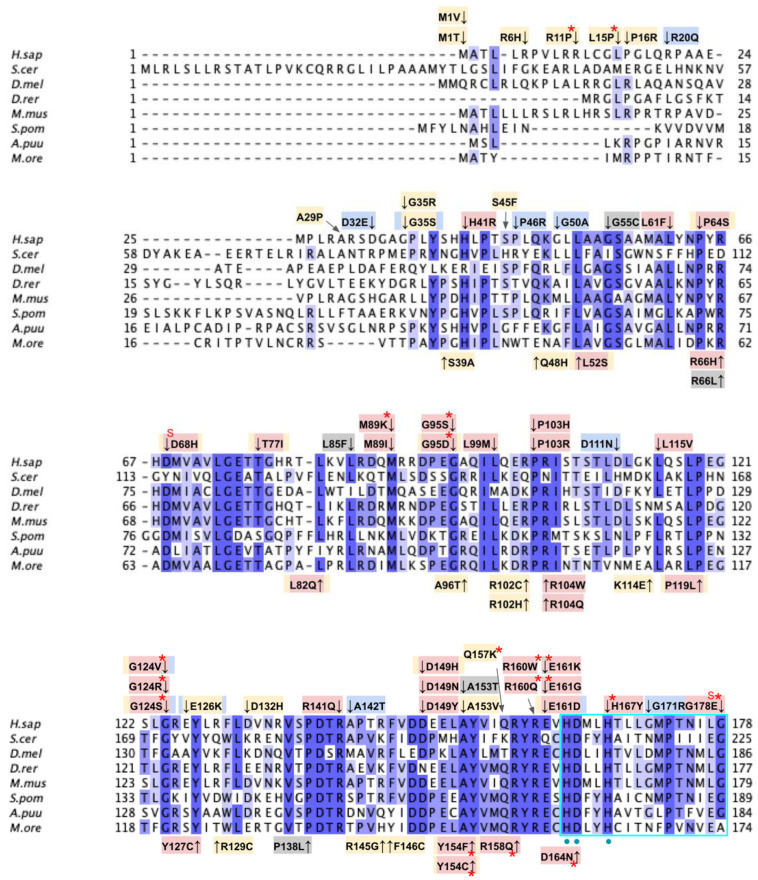

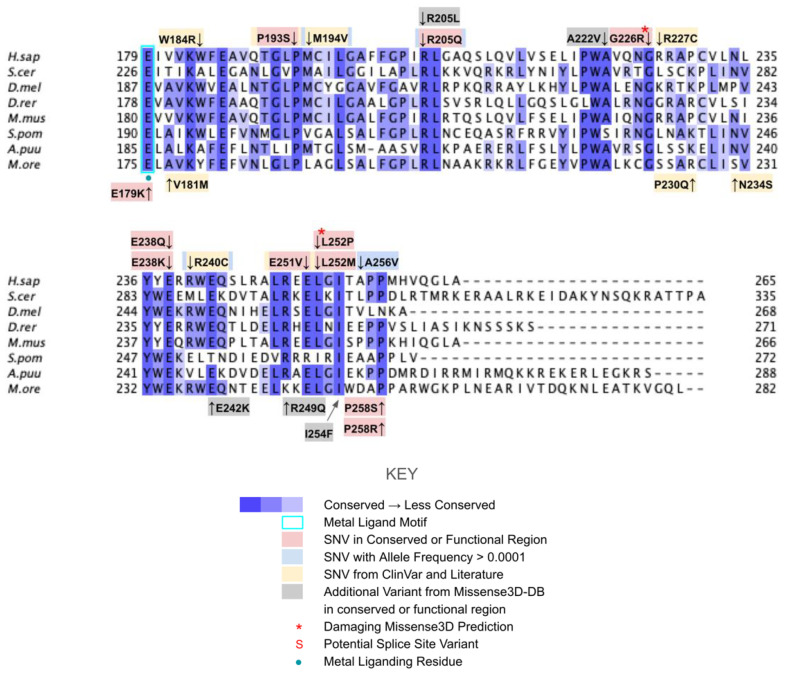
Labeled and annotated multiple sequence alignment of COQ4. Amino acid sequences of COQ4 were analyzed as described in Materials and Methods and include *Homo sapiens* (NCBI accession number NP_057119.3) and homologs in *Saccharomyces cerevisiae* (NP_010490.1), *Drosophila melanogaster* (NP_730270.1), *Danio rerio* (NP_001108192.1), *Mus musculus* (NP_848808.1), *Schizosaccharomyces pombe* (NP_593130.1)*, Aspergillus puulaauensis* (XP_041555109.1)*,* and *Marasmius oreades* (XP_043005754.1). See KEY for descriptions of the figure annotations. The metal ligand motif is boxed in cyan, and putative residues that ligand the metal ions are denoted by a blue dot, data from [47].

From gnomAD, ClinVar, Missense3D-DB, and literature, we identified 97 missense SNVs of interest (Figure 9; Supplemental Table S1). Of these variants, 60 coincided with a highly conserved region or functional residues, as determined from the multiple sequence alignment as well as structural studies with Alr8543. Of 22 variants classified as structurally damaging by Missense3D, 18 were contained within a highly conserved or functional region. From the ClinVar database and published research literature, 15 missense SNVs were determined to be either pathogenic or likely pathogenic. Four of these pathogenic or likely pathogenic variants were identified by Missense3D to be structurally damaging. Fifteen additional variants from gnomAD were included as frequent polymorphisms. Intriguingly, three of these frequent polymorphisms were identified in the research literature as deleterious. G124S (allele frequency of 1.13 × 10^−4^) has been identified as a founder mutation in the southern Chinese population [71]. Another, E161D (allele frequency of 6.03 × 10^−3^) was found in a patient who suffered from primary CoQ_10_ deficiency and later died from rhabdomyolysis [72,73]. A third, R240C (allele frequency of 1.79 × 10^−4^), occurred in a patient with progressive spasticity, motor impairment, and ataxia [74]. This patient harbored biallelic variants of COQ4 (P193S and R240C), and individual tests of these alleles of human COQ4 in yeast showed that neither variant could rescue a *coq4* yeast mutant [74].

**Figure 10:**
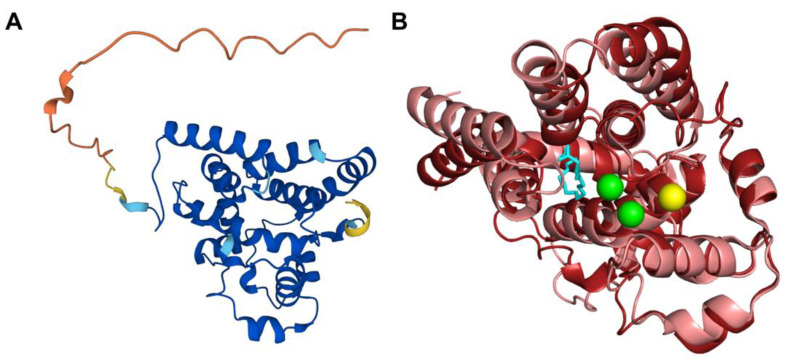
Comparison of the COQ4 AlphaFold model and the structure of Alr8543. (**A**) AlphaFold model of single chain of COQ4. Model color corresponds to model confidence as described in Figure 4. Figure was generated by the AlphaFold Protein Structure Database; adapted with permission from [39,54]. (**B**) Structural alignment of the human COQ4 AlphaFold model (shown in dark red) and Chain B of the crystal structure of Alr8543 protein (shown in salmon, PDB: 6E12) in complex with oleic acid (shown in cyan), Mg^2+^ (shown in green), and Cl^−^ (shown in yellow). Residues 1–44 of the AlphaFold model were omitted due to disordered structure.

The human COQ4 model was obtained from the AlphaFold Protein Structure Database (Figure 10A) [39,54]. The per-residue model confidence is shown in Figure 10A. A structural alignment of the human COQ4 AlphaFold model with the PDB 6E12 structure of the Alr8543 polypeptide, a COQ4 homolog from Nostoc sp., is shown in Figure 10B.

#### 3.2.1. SNVs in the Metal Liganding Motif

**Figure 11:**
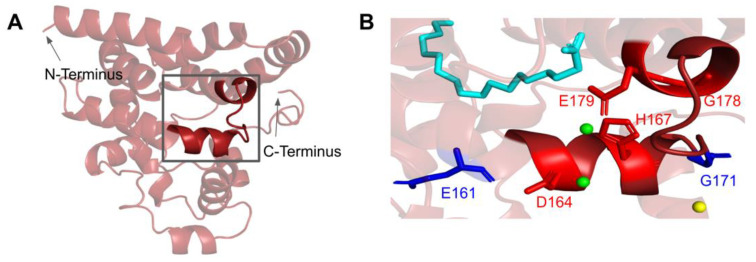
SNVs found in the putative COQ4 metal-liganding motif. (**A**) The metal-liganding motif (encompassing residues H163 to E179) is highlighted in red on the COQ4 model. (**B**) Locations of SNVs are depicted. Structural alignment of Alr8543 and COQ4 identifies the metal-liganding motifs in COQ4. Metal ions and oleic acid were obtained from this structural alignment. Residues are colored according to their corresponding SNVs in Figure 9. Mg^2+^ ions are designated as green spheres and Cl^−^ in yellow. Oleic acid is represented by the cyan structure.

**Figure A2:**
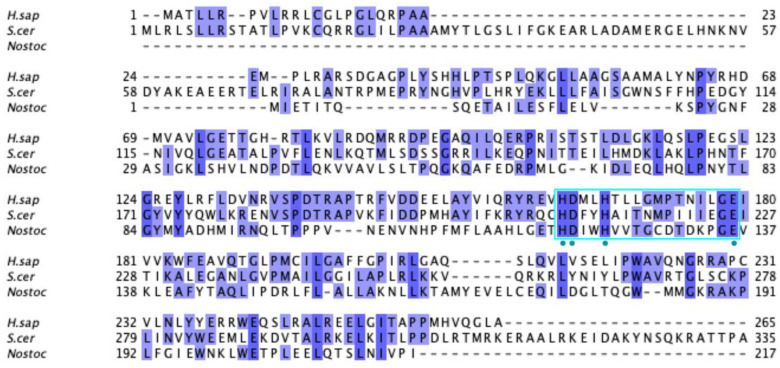
Multiple sequence alignment of *H. sapiens* COQ4, *S. cerevisiae* COQ4, and *Nostoc sp.* homolog Alr8543 (NCBI accession number: WP_010999537.1). Metal-liganding motif is boxed in cyan, and residues in Alr8543 (H120, D121, H124, and E136) that act to ligand the Mg^2+^ ions are marked with blue dots.

The amino acid sequence of COQ4 contains the highly conserved HDXXH-(X)_11_-E motif indicative of a metal ligand [47]. While the function and identity of the metal ligand remain unknown, it has been hypothesized that the ligand is sensitive to the redox state of mitochondria. Mutations in this region may destabilize coordination of the metal and CoQ synthome formation [47]. The motif spans residues H163 to E179 (Figure 11A). In the Alr8543 structure, the analogous E136 in the conserved HDXXH-(X)_11_-E motif ligands one Mg^2+^ ion (Figure A2). H124, the second H residue of the motif, forms an interaction with E. The D121 of this motif in Alr8543 ligands the second Mg^2+^ ion.

There are four SNVs reported in Missense3D-DB that occur at highly conserved residues within the metal-liganding motif: D164N, H167Y, G178E, and E179K (Figure 11B). Of these, D164, H167, and E179 correspond to residues predicted to ligand the metal ion. D164 is proposed to be analogous to the Mg^2+^ ion-liganding D121 in Alr8543 (Figure A2). H167 is proposed to be analogous to H120 in Alr8543, which stabilizes interactions with E136 that ligands the Mg^2+^ ion. E179 is proposed to be analogous to E136, which ligands the second Mg^2+^ ion in Alr8543 (Figure A2). A homologous point mutation of E179K in Coq4 of *S. cerevisiae* was unable to form the high molecular mass CoQ synthome [47]. Three SNVs in this region, D164N, H167Y, and G178E, were identified by Missense3D as being structurally damaging. None of these mutations have a reported clinical association on ClinVar.

The most common flags generated by Missense3D relate to the change or introduction of a buried charge, such as at D164 and H167. Such changes may destabilize the tertiary structure of COQ4 and may also affect the ability of COQ4 to interact with metal ligands.

#### 3.2.2. COQ4 Haploinsufficiency

Salviati et al. first identified the *COQ4* gene as being haploinsufficient [75]. This property is unusual because heterozygous carriers harboring a mutation in one of the other *COQ* genes are generally described as asymptomatic and produce normal levels of CoQ. *COQ4* haploinsufficiency was confirmed in both fibroblast and yeast cell models [75]. Intriguingly, one of the SNVs identified as a frequent polymorphism (E161D, Figure 11B) was detected as a heterozygous mutation in a patient with CoQ_10_ deficiency who had minor mental retardation and died of rhabdomyolysis [72,73]. Correction of the sequence variant in a cell culture model with CRISPR-mediated editing rescued the CoQ-deficient phenotype [73]. It is tempting to speculate that the haploinsufficiency of COQ4 may be related to its putative role in “scaffolding” the CoQ synthome, as proteins that serve analogous functions appear to be sensitive to changes in gene dosage [76].

### 3.3. COQ5

COQ5 is an AdoMet-dependent *C*-methyltransferase and a component of the CoQ synthome [18,77]. It is required for the conversion of DDMQH_2_ to DMQH_2_ in both *S. cerevisiae* and humans (Figure 1A) [18,78,79]. The human protein has been shown to rescue Coq5-deficient yeast that harbor stable but inactive Coq5 polypeptides, and it can also rescue *coq5* null mutants that overexpress Coq8 [18,80]. COQ5 is thought to be crucial for the stability of the synthome, as yeast lacking Coq5 are CoQ-less, respiratory-deficient, and do not form an intact synthome [59,80,81]. Partial knockdown of *COQ5* in human cells corroborates the findings observed in yeast *coq5* mutants and supports the crucial role of COQ5 in CoQ synthome formation and CoQ biosynthesis [20]. Crystal structures of the apo and AdoMet-bound yeast Coq5 dimers have been determined [48], revealing its seven β-strand Rossmann fold structure typical of the most common class I methyltransferases [64,82]. These crystal structures, in addition to known methyltransferase motifs [64], have helped elucidate the AdoMet and substrate-binding pockets as well as key residues involved in protein dimerization.

To date, only three patients with a mutation in *COQ5* have been documented. These related patients carried a 9590-bp duplication of the last four exons and part of the 3′ UTR of *COQ5*, resulting in an elongated 3′ UTR. They were deficient in CoQ_10_ and exhibited symptoms exclusive to the central nervous system, including encephalopathy, intellectual disability, ataxia, and cerebellar atrophy. The clinical outcome of long-term CoQ_10_ supplementation on these patients was equivocal [83]. Meanwhile, pathogenic SNVs in the *COQ5* gene have not been identified, although yeast studies have identified point mutations affecting the activity and stability of the Coq5 polypeptide, all of which resulted in respiratory deficiency [80].

**Figure 12:**
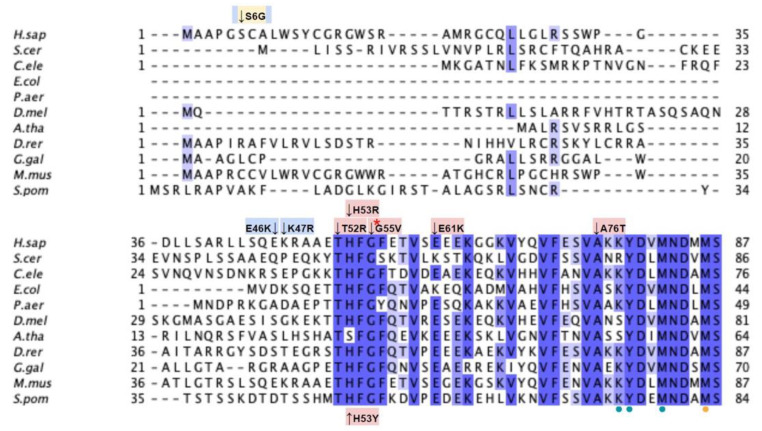

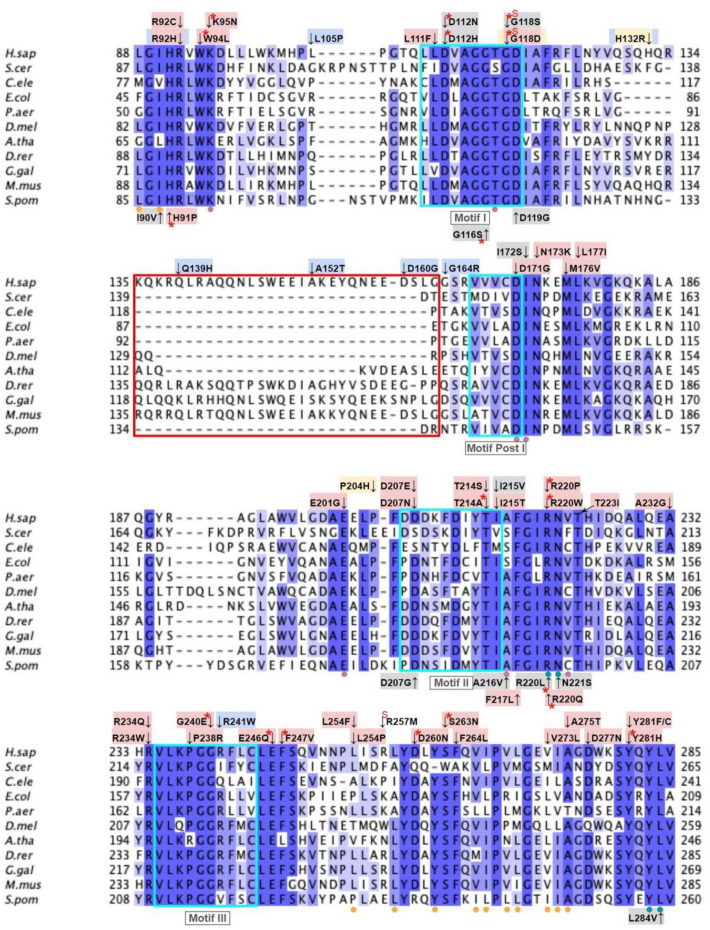

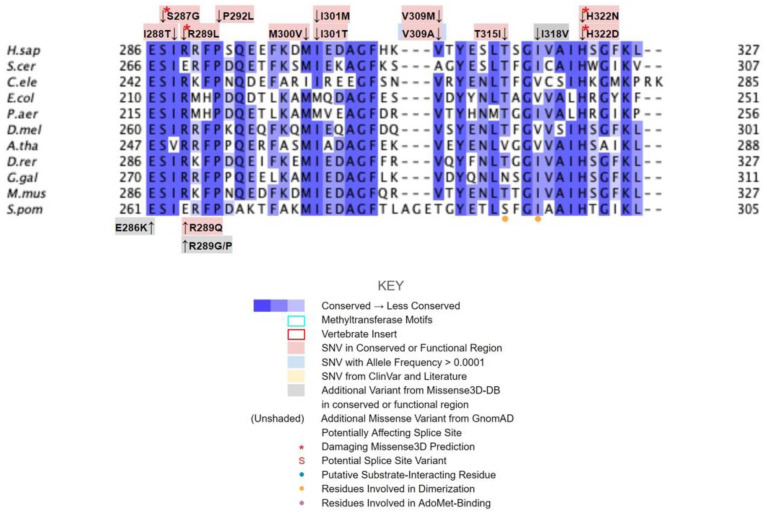
Labeled and annotated multiple sequence alignment of COQ5. Amino acid sequences of COQ5 were analyzed as described in Materials and Methods and include *Homo sapiens* (NCBI accession number NP_115690.3) and homologs in *Saccharomyces cerevisiae* (NP_013597.1), *Caenorhabditis elegans* (NP_498704.1), *Escherichia coli* UbiE (YP_026260.1), *Pseudomonas aeruginosa* UbiE (NP_253750.1), *Drosophila melanogaster* (NP_572865.1), *Arabidopsis thaliana* (NP_200540.1), *Danio rerio* (NP_001004541.1), *Gallus gallus* (NP_001006194.1), *Mus musculus* (NP_080780.1), and *Schizosaccharomyces pombe* (NP_587834.1). See KEY for descriptions of the figure annotations. Functional residues (dotted) were determined from the yeast Coq5 crystal structure (data from [48]). Methyltransferase motifs I, post-I, II, and III are boxed in cyan (data from [18]). An insert exclusively found in vertebrate species is boxed in red. Note that W243 in *S. cerevisiae* (aligned to S263 in the human sequence) is the residue involved in dimerization based on the crystal structure. However, since all other species in the multiple sequence alignment have a Tyr insertion at that position, Y262 was chosen as the functional residue instead due to its similar aromatic nature.

From gnomAD, ClinVar, and Missense3D-DB, we identified 88 missense SNVs of interest (Figure 12; Supplemental Table S1). Of these variants, 76 coincided with a highly conserved region or functional residue, which were determined from the multiple sequence alignment as well as structural studies on yeast Coq5 [48]. All 24 variants classified as structurally damaging by Missense3D were contained in this group. In addition, ten variants from gnomAD that did not occur in highly conserved or functional residues were included as frequent polymorphisms. None of these variants were predicted to be structurally damaging.

**Figure 13:**
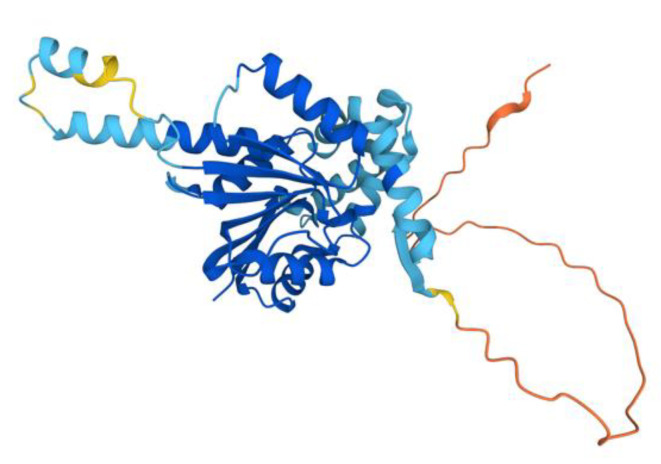
AlphaFold model of the COQ5 monomer. Model color corresponds to model confidence as described in Figure 4. Figure was generated by the AlphaFold Protein Structure Database; adapted with permission from [39,54].

**Figure 14:**
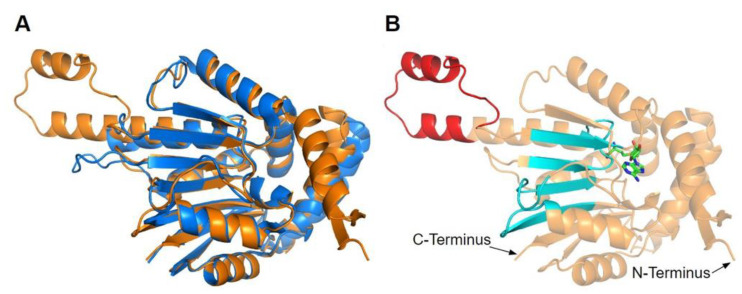
Alignment of AlphaFold-generated model of human COQ5 with existing yeast Coq5 crystal structures. (**A**) Superimposition of the human COQ5 model (shown in orange), adapted with permission from [39,54], and the crystal structure of AdoMet-bound yeast Coq5 (shown in blue, PDB: 4OBW). The first 47 residues of the AlphaFold model, which contains a disordered region with low confidence, were omitted for clarity. (**B**) Human COQ5 model with conserved methyltransferase motifs I-III and post-I highlighted in cyan and vertebrate insert in red. AdoMet (shown in green) was modeled via structural alignment with the crystal structure of AdoMet-bound yeast Coq5 using PyMOL.

**Figure A3:**
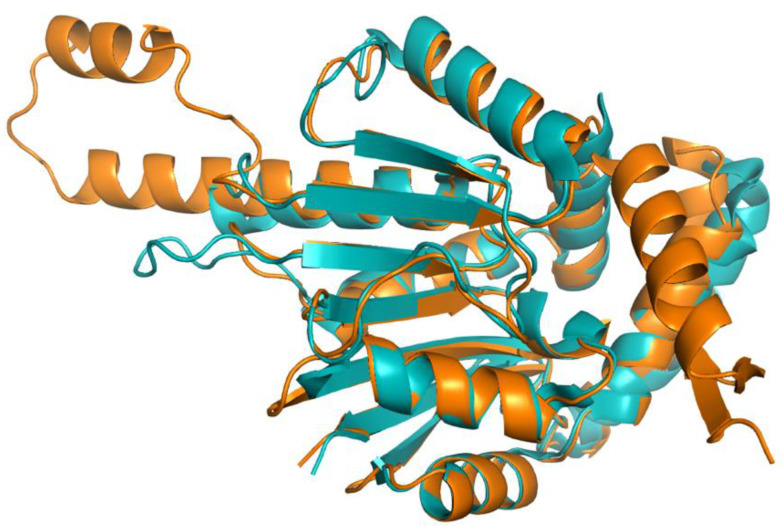
Structural alignment of the human COQ5 model (shown in orange) and the crystal structure of *S. cerevisiae* Coq5, apo form (shown in teal, PDB: 4OBX). Residues 1–47 of the AlphaFold model were omitted due to disordered structure. Alignment was performed using PyMOL.

The human COQ5 model was obtained from the AlphaFold Protein Structure Database (Figure 13) [39,54]. Compared to the crystal structure of AdoMet-bound yeast Coq5 (PDB: 4OBW) and apo yeast Coq5 (PDB: 4OBX), this model consisted of an additional α-helix N-terminal to the core structure, which was not visible in the yeast Coq5 crystal structure (Figure 14A and Figure A3) [48]. The α3 of the human COQ5 model is extended compared to that of the yeast Coq5 structure and is followed by a loop region containing a shorter helix. This region reflects an insert that spans residues 134–164, found exclusively in vertebrate species (Figure 12). The core seven β-strand structure, as well as the AdoMet and substrate-binding pockets, remain largely similar. In addition to their conserved three-dimensional structure, each of the four methyltransferase motifs (motifs I-III and post-I) in COQ5 approximately correspond to one β-strand connected to loops that make up the AdoMet binding site (Figure 14B) [64,65].

#### 3.3.1. SNVs in Methyltransferase Motifs I and Post-I

**Figure 15:**
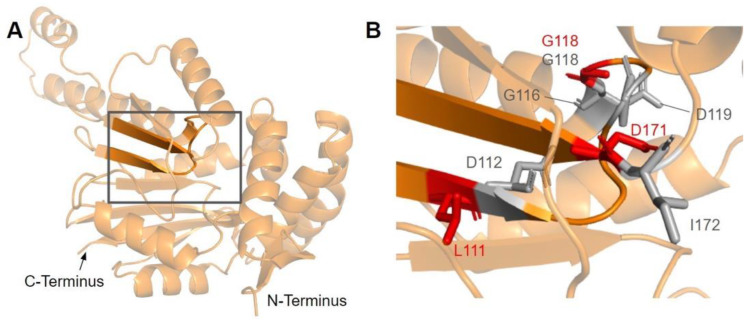
SNVs found in motif I and post-I of COQ5. (**A**) Motif I (lower β-strand, residues L110 to D119) and post-I (upper β-strand, residues V167 to D171) highlighted on the COQ5 model. The first 47 residues were truncated for simplicity. (**B**) Locations of SNVs in motifs I and post-I are depicted. Residues are colored according to their corresponding SNVs Figure 12.

The first of the four motifs, motif I, is a Gly-rich motif that makes up the first β-strand followed by an extended turn (Figure 15A) [65]. The homologous residue of T117 within this motif (S122 in yeast) has been shown to interact with the amino group of AdoMet in yeast Coq5, while the remaining residues line the AdoMet binding pocket (PDB: 4OBW) [48]. Mutations occurring in this region, which roughly corresponds to residues L110 to D119, may affect the binding of the AdoMet cofactor and therefore enzyme activity. In total, we identified seven SNVs of interest spanning five distinct amino acid positions in this motif, all of which occur on highly conserved residues (Figure 12 and Figure 15B). D112N, D112H, G116S, G118S, and D119G were reported in Missense3D-DB, and all but D119G were classified as structurally damaging by Missense3D. These point mutations may result in the loss of stabilizing interactions within the protein or with the cofactor, as well as the loss of buried Gly residues, which may destabilize the extended turn. L111F and G118D were reported in gnomAD, with G118D having an unknown clinical significance in ClinVar. G118D was classified as structurally damaging due to similar reasons as the previous group of variants, while L111F was neutral. In yeast, a G120R point mutant, homologous to G115 in human COQ5, was found to be structurally stable but catalytically inactive [80], further supporting the Missense3D classifications. However, it is worth noting that the two SNVs at position 118, G118S and G118D, flank the splice sites between exons 2 and 3 on the 5′ and 3′ ends, and may affect splicing in addition to protein structure.

After motif I, the second β-strand is partially composed of motif post-I, which contains a highly conserved acidic residue responsible for interacting with the hydroxyl groups on the ribose ring of AdoMet (Figure 15A) [48,65]. We have defined motif post-I as residues V167 to D171, although flanking residues I172 and N173 are also highly conserved. There are two particular SNVs of interest in the motif post-I region found in gnomAD, namely D171G at the conserved acidic residue and I172S adjacent to it, both of which extend into the AdoMet binding pocket and are annotated as AdoMet-binding (Figure 12 and Figure 15B). These mutations likely abolish interaction with the ribose ring of AdoMet, but were not classified as damaging by Missense3D due to the residues being solvent-exposed in the binding pocket of the model.

#### 3.3.2. SNVs in Methyltransferase Motifs II and III

**Figure 16:**
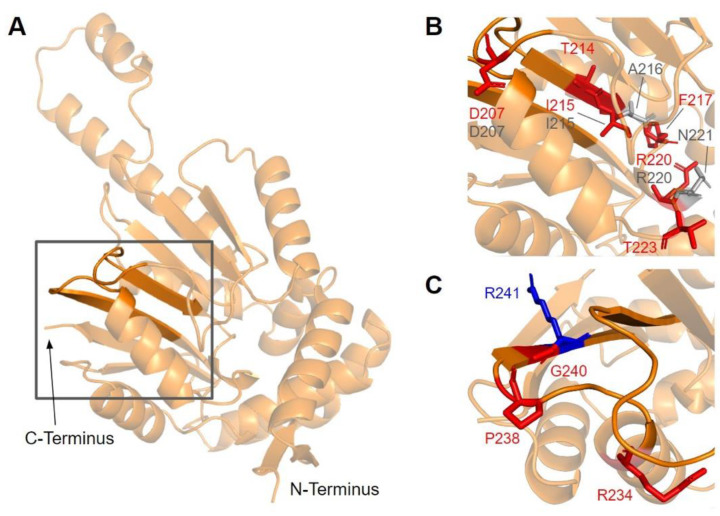
SNVs found in motifs II and III of COQ5. (**A**) Motif II (upper β-strand, residues D206 to I215) and motif III (lower β-strand, residues V235 to F242) highlighted on the COQ5 model. The first 47 residues were truncated for simplicity. (**B**,**C**) Locations of SNVs in motif II (**B**) and motif III (**C**) are depicted. Residues are colored according to their corresponding SNVs in Figure 12.

Motif II (D206 to I215) is located in the fourth β-strand (Figure 16A), where residues K209 to D211 are thought to make extensive contacts with motifs I and III to help align the methyltransferase domain [65]. Residues immediately after motif II form a loop that extends into the AdoMet and substrate-binding pockets. This loop region includes A216 and V222, which are homologous to yeast Coq5 residues interacting with AdoMet, as well as R220 and N221, which are thought to interact with the substrate, DDMQH_2_ [48]. In motif II and the loop that follows it, there are 15 SNVs of interest spanning eight distinct amino acid positions, all of which are highly conserved (Figure 12 and Figure 16B). D207G, A216V, A215V, R220L, and N221S were reported in Missense3D-DB, although all of them were classified as neutral by Missense3D except for R220L. From gnomAD, we identified variants D207E, D207N, T214S, T214A, I215T, F217L, R220P, R220W, R220Q, and T223I. Of these, T214A, R220P, R220W, and R220Q were classified as deleterious by Missense3D. The most common flags pertain to the loss of buried charges, residue-residue interactions, as well as a change in cavity size (R220). Cavity size likely refers to a change in the substrate binding pocket and is likely to result in a loss of activity. In yeast Coq5, the introduction of G199D (the residue homologous to G218 in human COQ5) resulted in a structurally stable but catalytically inactive mutant that was respiratory-deficient [80], suggesting that mutations in this region are likely pathogenic.

Motif III (V235 to C244) and the residues preceding it are located in a coil that becomes β-strand five (Figure 16A). The residues in this coil are responsible for aligning the residues in β5 and the active site [65]. From gnomAD, we identified five SNVs of interest spanning four distinct amino acid positions in this region (Figure 12 and Figure 16C). They are R234Q, R234W, P238R, G240E, and R241W, all of which are highly conserved, with the exception of R241W, which is a frequent polymorphism (allele frequency of 4.21 × 10^−4^). Only G240E was classified as damaging by Missense3D.

#### 3.3.3. SNVs on the Dimerization Interface

**Figure 17:**
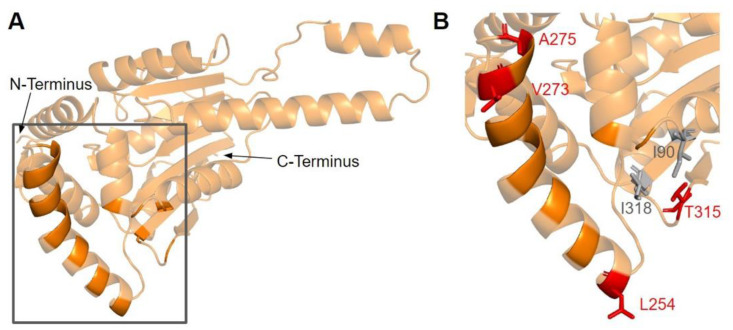
SNVs found in the COQ5 dimerization interface. (**A**) The dimerization interface of COQ5 largely consists of hydrophobic residues located on the α6 helix (left highlight). The first 47 residues were truncated for simplicity. (**B**) Locations of SNVs are depicted. Residues are colored according to their corresponding SNVs in Figure 12.

The dimerization interface, as determined from the crystal structures of the yeast Coq5 dimer (PDB: 4OBX and 4OBW), consists of hydrophobic residues homologous to M86, L88, I90, L254, L258, Y262, V266, I267, V269, L270, V273, I274, A275, T315, and I318 [48]. We identified a total of seven SNVs spanning six unique amino acid positions (Figure 17A). Among them, L254F, L254P, V273L, A275T, and T315I were found in gnomAD, while I90V and I318V were found in Missense3D-DB (Figure 17B). None of these variants were flagged as damaging by Missense3D, as the mutant residues were often hydrophobic. It is worth noting that all seven SNVs would appear as surface residues in the COQ5 model, which does not include an oligomeric structure. The effectiveness of Missense3D is limited in this region; thus, we have also provided the SIFT and Polyphen-2 classifications (Supplemental Table S1).

#### 3.3.4. Additional SNVs in Potentially Significant Regions

**Figure 18:**
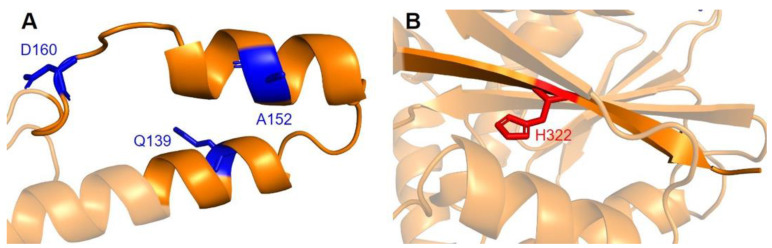
SNVs found in other potentially significant regions of COQ5. Locations of SNVs are depicted in (**A**) the vertebrate insert (left, residues K135 to G163) , and (**B**) in the C-terminus (right, residues V319 to L327). Residues are colored according to their corresponding SNVs in Figure 12.

Two additional regions on COQ5 are worth noting. The first is an insert roughly located at residues K135 to G163, which is found exclusively in vertebrate species. A BLAST search comprising the amino acid sequence of this region gave no significant hits [84]. Three SNVs with allele frequency larger than 1.00 × 10^−4^ are found in this region: Q139H, A152T, and D160G (Figure 18A). A152T was estimated to have an allele frequency of 1.18 × 10^−1^ by gnomAD, the most common among all missense SNVs found in the *COQ5* coding region. While none of these variants were classified as structurally damaging by Missense3D, this region may have evolved new functions, such as protein-protein interactions, that may be affected by these mutations. The second region of interest is the C-terminus of COQ5. Previous studies in yeast have identified a Coq5 point mutant located four residues away from the C-terminus. This mutant resulted in a partially active enzyme that resulted in a decrease in the steady-state levels of Coq3, Coq4, and Coq5, as well as respiratory deficiency [80]. The underlying cause of this phenotype is unclear, although it does suggest that the C-terminus of COQ5 may play a key role in stabilizing Coq5, whether it be through residue-residue interactions or some other means. Two SNVs, H322N and H322D, are found in this region, located just six amino acids away from the C-terminus (Figure 18B). Residue H322 was predicted to interact with Q294 by Missense3D. Both variants were classified as damaging, for reasons related to the loss of buried charges and hydrogen-bonding interactions.

### 3.4. COQ6

COQ6 is characterized as a class A flavoprotein monooxygenase with a tightly bound FAD cofactor [33,85,86]. However, unlike typical flavin-dependent monooxygenases which receive electrons directly via an NAD(P)H coenzyme, NAD(P)H instead delivers electrons to yeast Coq6 via ferredoxin Yah1 (FDX1 and FDX2 are human homologs) and ferredoxin reductase Arh1 (FDXR is a human homolog) [13]. It is not yet known if human homologs FDX1, FDX2, and FDXR perform the same electron transfer roles for COQ6. Coq6 is peripherally associated with the IMM and is responsible for the addition of a hydroxyl group to the C5 position on the CoQ ring precursor, converting HPB to DHPB (Figure 1A) [33]. While both humans and yeast use 4-HB as a ring precursor for CoQ biosynthesis, yeast cells also utilize para-aminobenzoic acid (pABA) as a ring precursor to CoQ [87]. Ozeir et al. showed that yeast Coq6, in addition to hydroxylating C5, is also able to deaminate the ring C4 position on the intermediate derived from pABA [88]. Yeast *coq6* null-mutants accumulate 3-hexaprenyl-4-hydroxybenzoic acid (HHB) and 3-hexaprenyl-4-aminobenzoic acid (HAB) [13,89]. Yeast *coq6* point mutants that express a stable but inactive Coq6 polypeptide accumulate 3-hexaprenyl-4-hydroxyphenol (4-HP_6_) [33]. The equivalent intermediate, 4-HP_10_, has also been observed to accumulate in human cells harboring a *COQ6* disruption [34]. This finding indicates that the C1-decarboxylation and C1-hydroxylation steps can occur prior to the C5-hydroxylation step (Figure 1A). Resolving the order of steps will require an in vitro assay for Coq6 and would also benefit from identification of the enzyme(s) that mediate the decarboxylation and hydroxylation at C1. Expression of human COQ6 partially complements yeast lacking Coq6, demonstrating the usefulness of *S. cerevisiae* as a model organism to study human *COQ6* mutations [90].

Mutations in the human *COQ6* gene are associated with primary CoQ_10_ deficiency and are further associated with several disease states, predominantly steroid-resistant nephrotic syndrome (SRNS) and sensorineural hearing loss (SNHL) [29,91,92,93]. Importantly, supplementation with either 3,4-dihydroxybenzoic acid (3,4-diHB) or vanillic acid (VA) partially restores CoQ biosynthesis in yeast *coq6* null mutants expressing human *COQ6* genes harboring pathogenic mutations; such a bypass of the C5-hydroxylation step with VA or 3,4-diHB also occurs when yeast coq6 null mutants overexpress Coq8 [33,90]. Supplementation with VA may provide a potential avenue of treatment for patients with CoQ_10_ deficiency due to mutations in the *COQ6* gene. However, clinical trials are required to determine both the safety and efficacy of this “bypass” therapy.

Drovandi et al. list 48 patients who likely have pathogenic mutations in COQ6 [91]. While mutations in *COQ6* are rare, they result in severe disease phenotypes across multiple physiological systems. A report by Perrin et al. detailed a case study of a Turkish patient born to consanguineous parents with a missense mutation in *COQ6* [94]. The patient presented with end-stage renal failure at five years old and subsequently received an organ transplant. At 17 years old, the patient presented with sudden loss of vision. Genetic analysis of the *COQ6* gene revealed the presence of the SNV A353D. The younger brother of this patient harbored the same homozygous mutation and presented with primary CoQ_10_ deficiency with SRNS and SNHL. Replacement therapy with idebenone (a hydrophilic short-chain CoQ_10_ analog) began at an earlier age for the second patient. Doimo et al. expressed the human A353D mutation, amongst others, in *S. cerevisiae coq6* at the corresponding residue A361 [90]. Results indicated that the mutation was hypomorphic, as yeast were viable but showed decreased growth on medium containing a nonfermentable carbon source. A recent study indicates that clusters of the A353D occur in Kazak, Turkish, and Iranian populations, and show a comparatively later disease onset [91]. In contrast, patients with a variant that is predominant in the Middle East (G255R), present with early disease onset, severe phenotype, and higher odds of mortality [91]. Further mutagenesis studies using yeast can help to elucidate the effects of mutations found in patients. In addition to the 13 known pathogenic mutations in *COQ6*, there have also been several reported heterozygous SNVs that may confer deleterious consequences.

**Figure 19:**
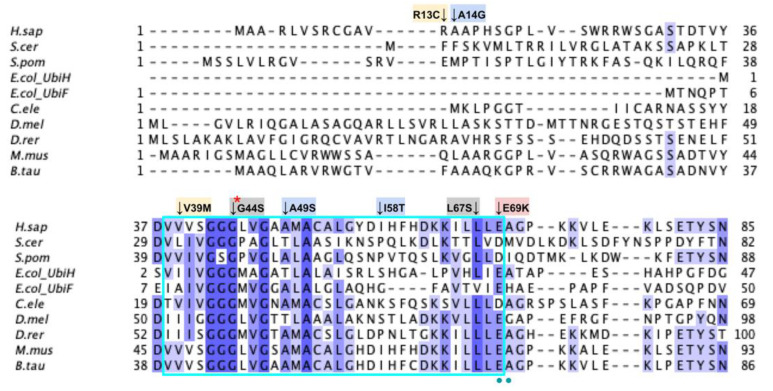

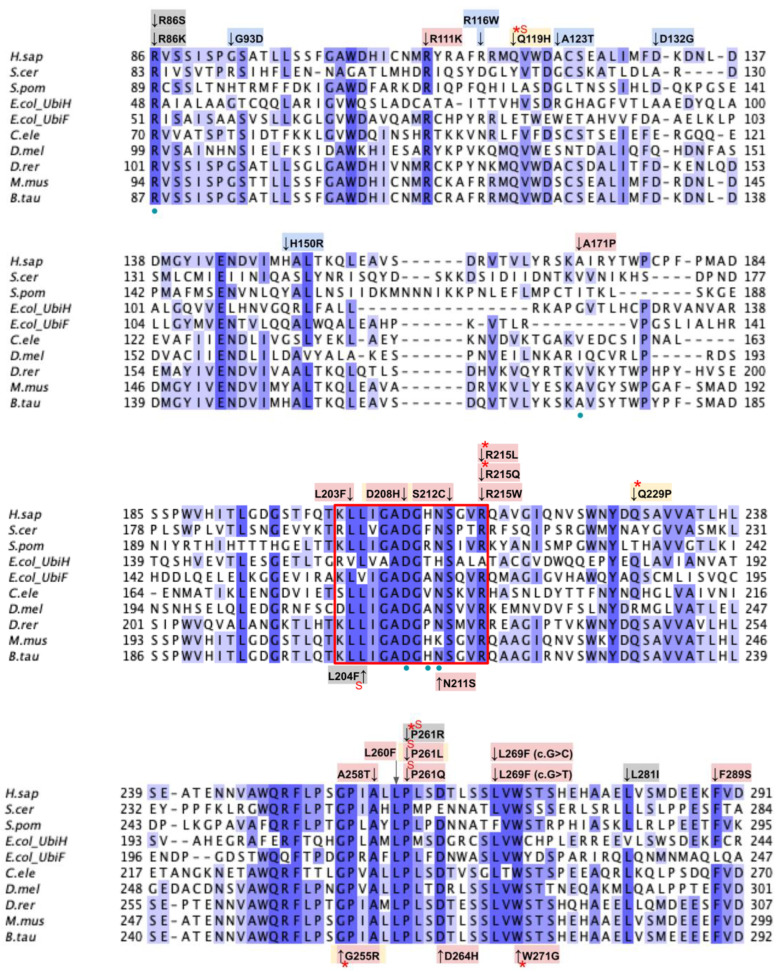

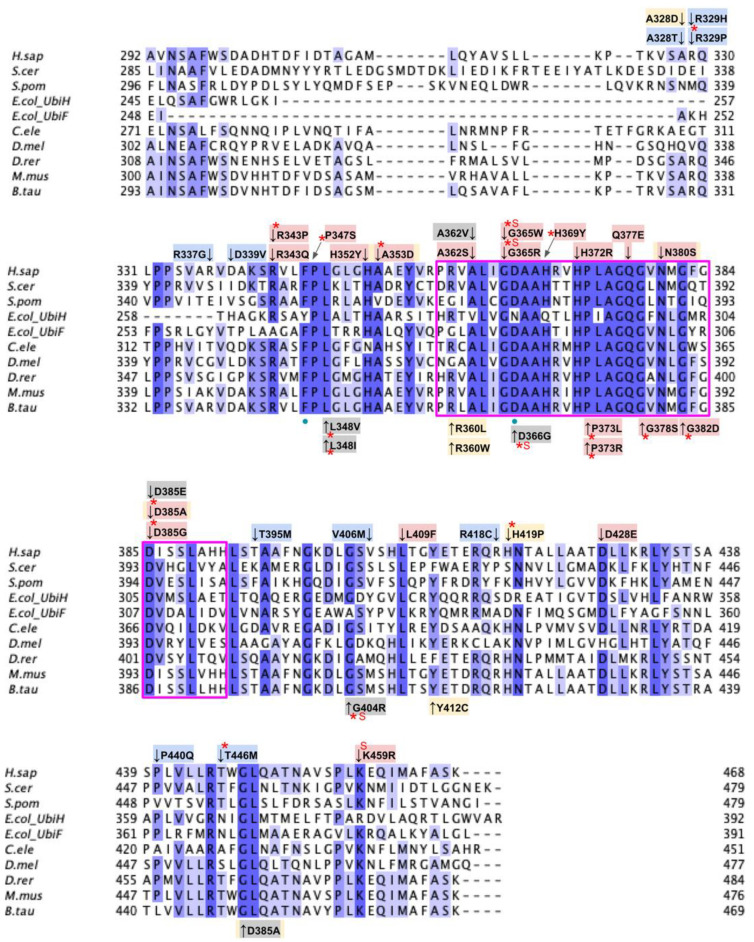

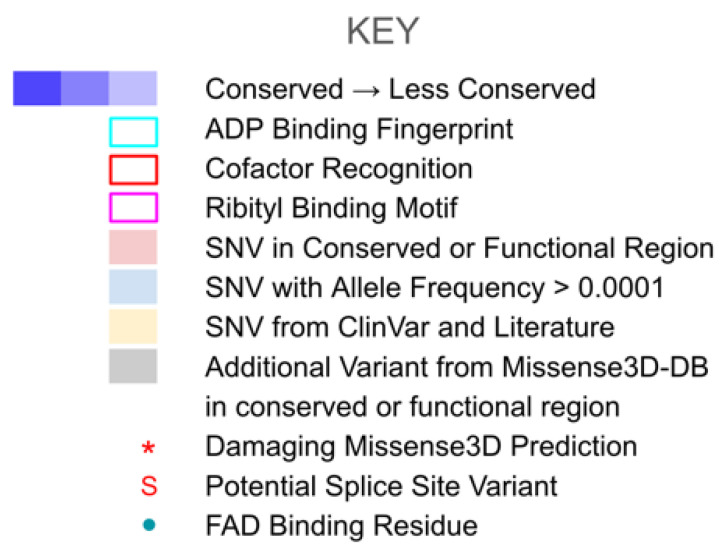
Labeled and annotated multiple sequence alignment of COQ6. Amino acid sequences of COQ6 were analyzed as described in Materials and Methods and include *Homo sapiens* (NCBI accession number NP_872282.1) and homologs in *Saccharomyces cerevisiae* (NP_011771.1), *Schizosaccharomyces pombe* (NP_595401.2)*, Escherichia coli* UbiH (NP_417383.1)*, Escherichia coli* UbiF (NP_415195.1)*, Caenorhabditis elegans* (NP_505415.2), *Drosophila melanogaster* (NP_608934.1), *Danio rerio* (NP_001038869.1), *Mus musculus* (NP_766170.2), and *Bos taurus* (NP_001039558.1). See KEY for descriptions of the figure annotations. The ADP Binding Fingerprint is boxed in cyan, the Cofactor Recognition motif is boxed in red, and the Ribityl Binding motif is boxed in magenta, data from [89]. FAD-binding residues are marked with blue dots, data from [49].

From gnomAD, ClinVar, Missense3D-DB, and literature we identified 82 missense SNVs of interest (Figure 19; Supplemental Table S1). Of these variants, 55 SNVs coincided with a highly conserved or functional residue, determined from the multiple sequence alignment and from structural studies on yeast, respectively [49]. Identification of functional residues was performed by in silico prediction of substrate-access tunnels and protein-cofactor interactions [49]. Residues predicted to be functional were subsequently corroborated by site-directed mutagenesis and in vivo functional assays in the yeast model [49]. Of the 27 variants classified as structurally damaging by Missense3D, 21 overlapped with the group of highly conserved or functional residues (Figure 19). From the ClinVar database, seven missense variants were determined to be pathogenic or likely pathogenic. Of those seven variants, three were identified by Missense3D to be structurally damaging. There were 18 SNVs in gnomAD identified as frequent polymorphisms. Intriguingly, one of the frequent polymorphisms T446M (allele frequency of 1.70 × 10^−4^) was identified as being structurally damaging by Missense3D.

**Figure 20:**
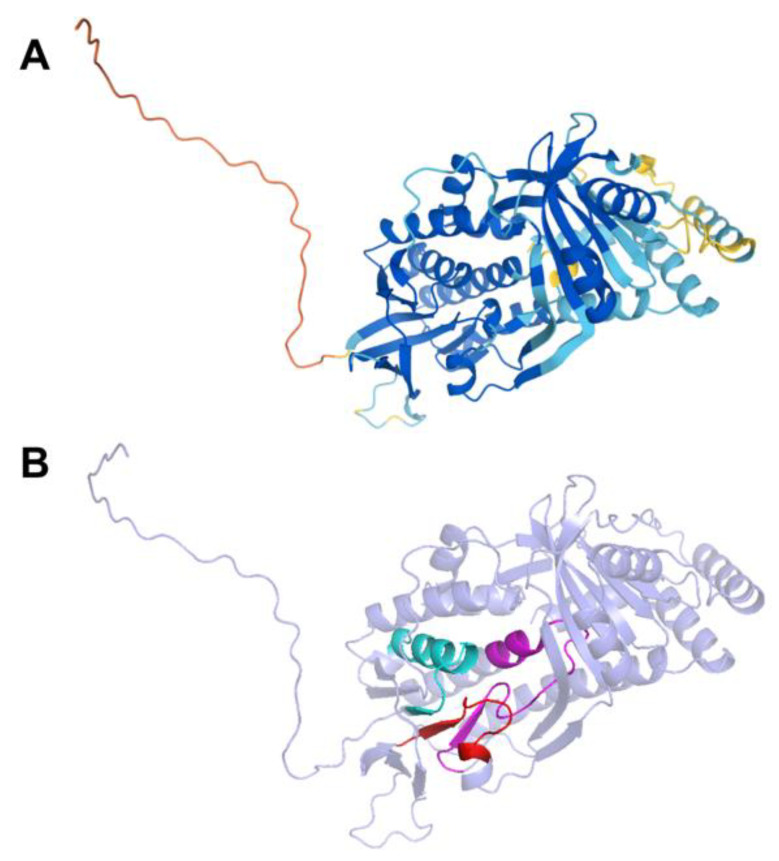
AlphaFold model of single chain of COQ6. (**A**) Model color corresponds to model confidence as described in Figure 4. Figure was generated by the AlphaFold Protein Structure Database [39,54]. (**B**) Structural motifs shown in Figure 19 are highlighted.

The human COQ6 model was obtained from the AlphaFold Protein Structure Database (Figure 20) [39,54].

#### 3.4.1. SNVs in the ADP-Binding Fingerprint

**Figure 21:**
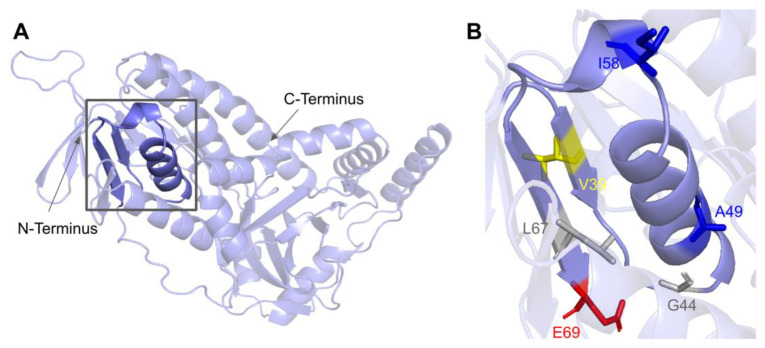
SNVs in the COQ6 ADP-binding fingerprint. (**A**) The ADP-binding βαβ-fold is highlighted on the COQ6 model and includes residues D37 to E69. Residues 1–35 have been truncated. (**B**) Locations of SNVs are depicted. Residues are colored according to their corresponding SNVs in Figure 19.

The COQ6 amino acid sequence contains the characteristic GXGXXG fingerprint necessary for binding the ADP-moiety of the FAD cofactor, along with other conserved residues involved in FAD/NAD^+^ recognition [89,95]. The segment forms a characteristic βαβ-fold that binds FAD, which is reflected in the COQ6 multiple sequence alignment and model (Figure 19 and Figure 21A). The βαβ-fold occurs near the N-terminus of COQ6 from residues D37 to E69 (Figure 21A). In this domain, we encounter six SNVs with relevance to this analysis. Mutations occurring in this region may affect COQ6 cofactor-binding capabilities. Two SNVs reported in Missense3D-DB occur at highly conserved residues within the ADP-binding fingerprint: G44S and L67S (Figure 21B). Of these two, G44S was identified by Missense3D as being structurally damaging, due to the replacement of Gly at a bend. Neither mutation has a clinical association listed on ClinVar. In this same motif gnomAD reports a SNV that occurs at a residue identified as FAD-binding: E69K (Figure 21B). This variant is of particular interest because of its potential to disrupt cofactor binding. However, E69K does not have any clinical association with primary CoQ_10_ deficiency, nor is it predicted to be structurally damaging by Missense3D. Two SNVs reported in gnomAD are identified as frequent polymorphisms: A49S and I58T (Figure 21B). Neither of these variants occurs at highly conserved residues. According to the ClinVar database, A49S has been listed as a benign mutation. There is no ClinVar annotation for I58T. Both of these variants are characterized as neutral by Missense3D. One additional SNV is reported in ClinVar in this region: V39M (Figure 21B). This variant has an uncertain significance label on ClinVar and is characterized as structurally neutral by Missense3D.

#### 3.4.2. SNVs in the Cofactor Recognition Structure

**Figure 22:**
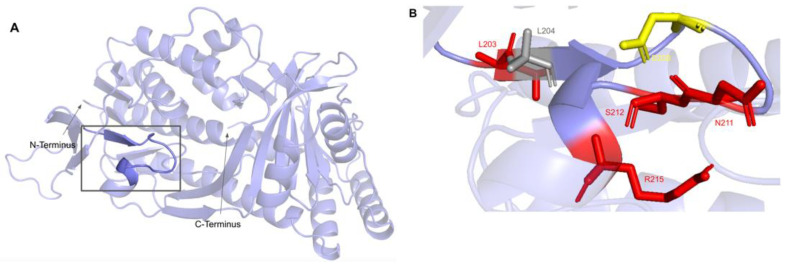
SNVs found in the COQ6 NAD(P)H/FAD recognition sequence. (**A**) NAD(P)H/FAD recognition structure highlighted on COQ6 model, residues K203 to R215. Residues 1–35 have been truncated. (**B**) Locations of SNVs are depicted. Residues are colored according to their corresponding SNVs in Figure 19.

This second region on the COQ6 polypeptide is suggested to recognize the pyrophosphate moiety of the FAD cofactor [89]. The cofactor recognition structure occurs from residues K202 to R215 (Figure 22A). In this domain, we encounter eight SNVs with relevance to this analysis. Mutations in functional residues contributing to FAD-binding are predicted to diminish COQ6 function, resulting in a deficiency in CoQ_10_. There are six SNVs reported in gnomAD that affect highly conserved residues within this region: L203F, N211S, S212C, R215L, R215Q, and R215W (Figure 22B). Of these six variants, R215L and R215Q were reported as structurally damaging by Missense3D. N211S may disrupt catalytic function, as it occurs at a residue identified as FAD-binding and is flagged by Missense3D as a buried charge replaced. However, this mutation does not have any clinical association. There is one additional SNV reported in Missense3D-DB occurring at a highly conserved residue: L204F (Figure 22B). This mutation is predicted to be structurally neutral by Missense3D. L204F does not have a clinical association with CoQ_10_ deficiency. ClinVar reports one SNV in this region: D208H (Figure 22B). This variant occurs at a highly conserved residue identified as FAD-binding and has a clinical association of unknown significance. This mutation is reported to lead to haploinsufficiency of COQ6, predisposing individuals harboring the mutation to schwannomatosis, which is a rare genetic disorder resulting in multiple benign schwannomas growing on peripheral nerves [96]. However, further studies must be conducted to confirm this link [97].

#### 3.4.3. SNVs in the Ribityl Binding Motif

The third region of known importance on the COQ6 polypeptide has been implicated in binding to the ribityl moiety on FAD. It contains the consensus fingerprint for ribityl binding, including the conserved Asp necessary to hydrogen bond with O-3 of the ribityl moiety [89,98]. This region is predicted to reside near the putative catalytic region containing the other amino acid fingerprints necessary for FAD-binding. Mutations in functional residues contributing to coenzyme binding are predicted to diminish COQ6 function, resulting in a deficiency in CoQ_10_.

**Figure 23:**
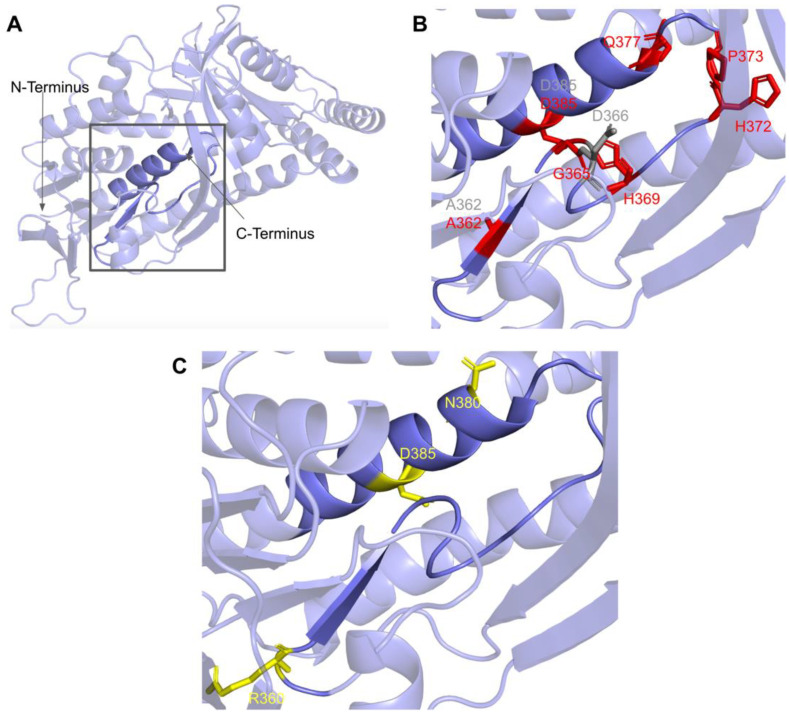
SNVs found in the COQ6 ribityl binding motif. (**A**) Ribityl binding motif is highlighted on the COQ6 model, from residues P359 to H392. Residues 1–35 have been truncated. (**B**) Locations of SNVs in conserved or functional regions are depicted. (**C**) Locations of ClinVar and literature variants are depicted. Residues are colored according to their corresponding SNVs in Figure 19.

This region spans residues P359 to H392 (Figure 19 and Figure 23A). In this domain, we encounter 18 SNVs relevant to this analysis. There are 11 SNVs reported in gnomAD that occur in highly conserved residues within this region. Eight of these 11 variants, G365W, G365R, H369Y, P373L, P373R, G378S, G382D, and D385G are reported to be structurally damaging by Missense3D. The high number of variants predicted to be structurally damaging in this region may be indicative of its structural importance to COQ6 integrity. Three additional SNVs are reported in Missense3D-DB to occur at highly conserved residues: A362V, D366G, and D385E (Figure 23B). Of the three, only D366G is predicted to be structurally damaging by Missense3D. This variant is also located at the conserved Asp predicted to bind FAD and may therefore disrupt COQ6 function. None of these mutations have clinical associations.

In this region, four SNVs are reported in ClinVar and the research literature: R360L, R360W, N380S, and D385A (Figure 23C). R360L is associated with primary CoQ_10_ deficiency and is labeled as likely pathogenic. R360W was found in a compound-heterozygous patient with the frameshift c.804delC mutation and was determined to be pathogenic [99]. Patients homozygous for R360W have been identified in China and Central/Eastern Europe and have been associated with a higher risk of cardiomyopathy, growth retardation, and neurologic involvement [91]. The N380S variant does not have a condition associated with it but is labeled as likely pathogenic by ClinVar. The D385A variant is reported and has been predicted in silico to be deleterious but has not been associated with any clinical manifestations [100]. This D385A variant is the only one of these four SNVs that is predicted to be structurally damaging by Missense3D.

The most common flags generated by Missense3D for this region concern the change of buried residues. The most consequential changes pertain to the replacement of buried charges and Gly residues. Such variants have the potential to disrupt FAD-binding and protein folding.

### 3.5. COQ7

COQ7 is characterized as a di-iron carboxylate hydroxylase [13]. It is peripherally associated with the matrix side of the IMM and is responsible for the penultimate step of CoQ biosynthesis, hydroxylating DMQH_2_ to DMeQH_2_ [101,102]. Addition of 2,4-diHB was shown to restore synthesis of CoQ_6_ in the yeast *coq7* null mutant when Coq8 was overexpressed [59]. The overexpression of Coq8 stabilizes the yeast Coq polypeptides that are otherwise degraded in the *coq* null mutants. The use of an alternate ring precursor to “bypass” the blocked Coq7 hydroxylase step in CoQ biosynthesis is similar to the previously described VA bypass of certain yeast *coq6* point mutants, or the *coq6* null mutant overexpressing Coq8 [33,90]. Tests of 2,4-diHB bypass therapy in patient fibroblasts harboring *COQ7* mutations indicated that such a bypass was not uniformly successful and seemed to have the most benefit in cells with a more profound deficiency in COQ7 [103]. Hence, the outcome of such bypass therapies is likely to be quite dependent on the nature of the mutations.

Human COQ7 is able to complement yeast lacking Coq7, demonstrating conserved functionality across species as well as the viability of *S. cerevisiae* as a model organism to study human *COQ7* mutations [104]. Mutations in the human *COQ7* gene are associated with primary CoQ_10_ deficiency and are further associated with several disease states, predominantly hypertonia and sensorineural hearing loss (SNHL) [29].

**Figure 24:**
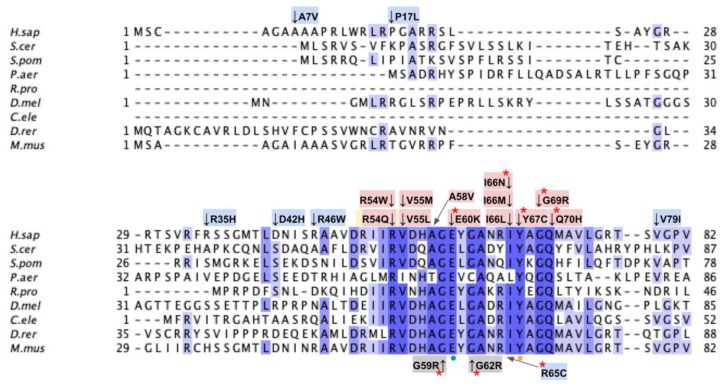

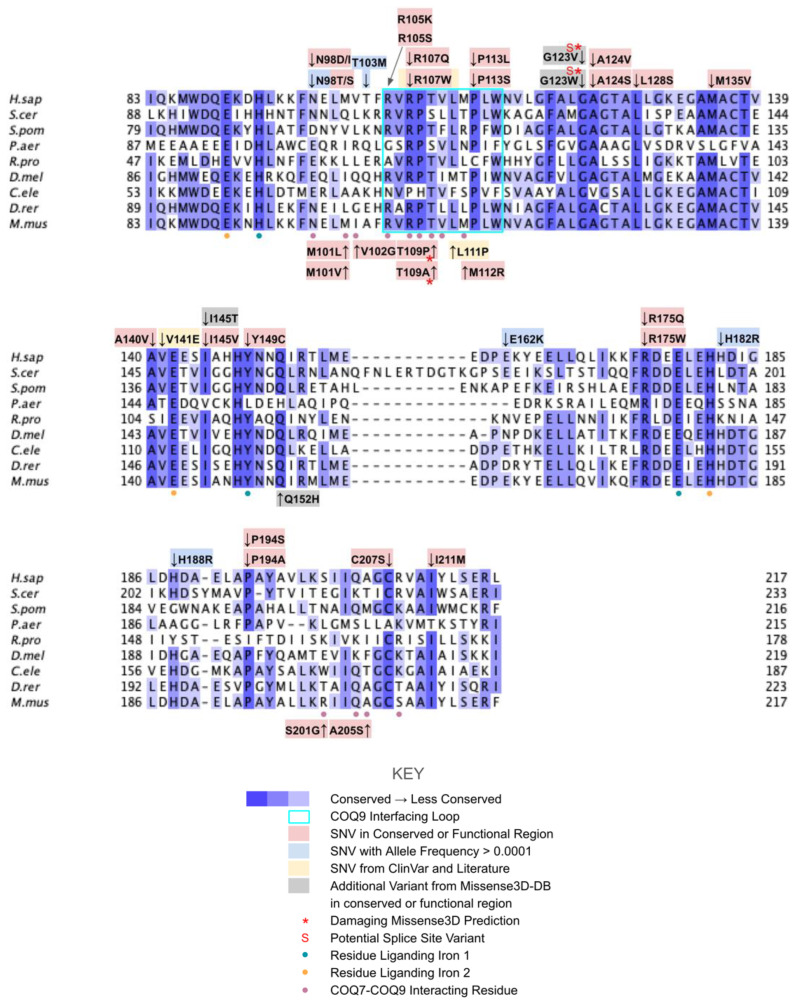
Labeled and annotated multiple sequence alignment of COQ7. Amino acid sequences of COQ7 were analyzed as described in Materials and Methods and include *Homo sapiens* (NCBI accession number NP_057222.2) and homologs in *Saccharomyces cerevisiae* (NP_014768.2), *Schizosaccharomyces pombe* (NP_595416)*, Pseudomonas aeruginosa* (QLJ91605.1)*, Rickettsia prowazekii* (ADE29699)*, Drosophila melanogaster* (NP_651967.2), *Caenorhabditis elegans* (NP_498128.1), *Danio rerio* (NP_001076480.1), and *Mus musculus* (NP_034070.1). See KEY for descriptions of the figure annotations. The COQ9 interfacing loop is boxed in cyan, data from [50]. Residues that ligand iron 1 are marked with blue dots, and those that ligand iron 2 are marked with orange dots, data from [52]. COQ7-COQ9 interacting residues are marked with purple dots, data from [50,52].

From gnomAD, ClinVar, Missense3D-DB, and literature we identified 60 missense SNVs of interest (Figure 24; Supplemental Table S1). Of these variants, 47 coincided with a highly conserved region or functional residue, which were determined from the multiple sequence alignment as well as structural studies on human COQ7 [50,52]. Eleven of the twelve variants classified as structurally damaging by Missense3D were contained in this group (Figure 24). From the ClinVar Database and literature, four missense variants were determined to be pathogenic or likely pathogenic. None of these four variants were identified by Missense3D to be structurally damaging. There were 13 SNVs in gnomAD identified as frequent polymorphisms. One of these, R65C (allele frequency of 2.76 × 10^−4^) was identified as being structurally damaging by Missense3D.

**Figure 25:**
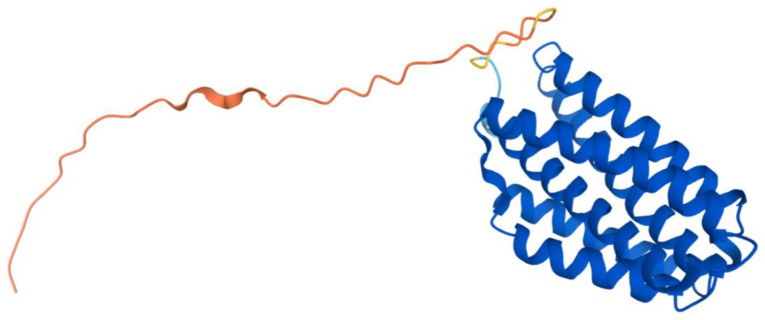
AlphaFold model of single chain of COQ7. Model color corresponds to model confidence as described in Figure 4. Figure was generated by the AlphaFold Protein Structure Database [39,54].

The human COQ7 model was obtained from the AlphaFold Protein Structure Database (Figure 25) [39,54]. The structure of a human COQ7:COQ9 complex was recently determined (PDB: 7SSS and 7SSP) [52].

#### 3.5.1. SNVs in a Loop of COQ7 That Interfaces with COQ9

**Figure 26:**
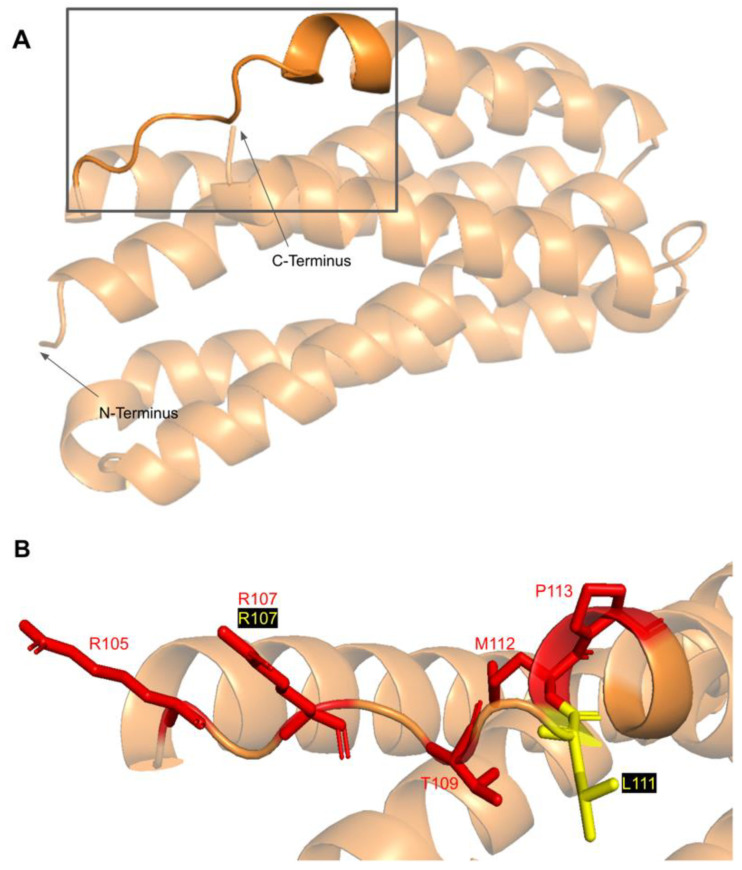
SNVs found in the COQ7 loop that interfaces with COQ9. (**A**) The loop (residues R105 to W115) that interfaces with COQ9 are highlighted on the COQ7 model. Residues 1–43 have been truncated. (**B**) Locations of SNVs are depicted. Residues are colored according to their corresponding SNVs in Figure 24.

The COQ7 polypeptide contains a loop (residues R105 to W115) that is situated between α-helix 1 and α-helix 2 and is composed predominantly of residues with hydrophobic side-chains. This loop was predicted to interact with COQ9 [50] and was recently observed at the interface between COQ7 and COQ9 in the structure determined for an octameric COQ7:COQ9 complex [52]. The residues in this loop are situated on the outer face of COQ7 (Figure 26A). In this domain, we encounter six SNVs with relevance to this analysis. Mutations in this region have the potential to decrease COQ7-COQ9 hydrophobic, hydrogen-bonding, and salt bridge interactions noted by [52]. There are nine SNVs reported in gnomAD occurring at highly conserved or functional residues in this region (Figure 26B). Of these nine SNVs from gnomAD, only variants at T109 were flagged by Missense3D as structurally damaging (buried H-bond breakage). None of these variants are present as homozygotes in gnomAD, nor are they associated with a deficiency in CoQ_10_. The variant R107W was identified in ClinVar as having uncertain significance and occurs at a highly conserved residue (Figure 26B). This variant is adjacent to P108, a key residue predicted to interact with COQ9 [50]. Missense3D characterizes R107W as neutral. L111P was identified as a homozygous mutation in a six-year-old girl presenting with SNHL and spasticity by Wang et al. (Figure 26B) [103]. However, the severity of this mutation was determined to be dependent on the presence of another variant, T103M, which is identified here as a frequent polymorphism (allele frequency of 6.24 × 10^−1^). Missense3D characterizes T103M as neutral.

#### 3.5.2. Residues near the C-terminus of COQ7 That Form Contacts with COQ9

The COQ7:COQ9 complex co-crystallized with a core of phospholipids, suggesting that membrane association is crucial for complex formation [52]. Four residues located in the C-terminal α-helix 6 make additional contacts with COQ9: S201, Q204, A205, and R208. It seems possible that the variant C207S, nestled among these residues and occurring at a highly conserved Cys, might impact the association of COQ7 with COQ9. There are also two variants that occur at reported interacting residues: S201G and A205S. Both variants are characterized by Missense 3D as neutral but may have the potential to disrupt COQ7:COQ9 interaction.

#### 3.5.3. SNVs in the Predicted Iron-Liganding Motif

**Figure 27:**
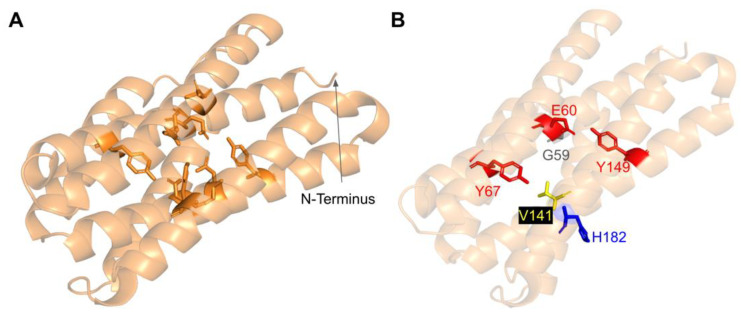
SNVs found in COQ7 residues that are adjacent to or that ligand the iron atoms. (**A**) The residues predicted to ligand the Fe (II) atoms are depicted in orange on the COQ7 model; E60, Y67, E90, H93, E142, Y149, E178, and H181. (**B**) Locations of SNVs are depicted. Residues are colored according to their corresponding SNVs in Figure 24. Residues 1–43 have been truncated in both panels.

COQ7 was first identified as a di-iron protein with the motif E X_n1_ EXXH X_n2_ E X_n3_ EXXH, where n represents a variable number of connecting residues [51]. These residues form carboxylate ligands that are predicted to bind to two Fe (II) atoms located within the four-helix bundle of COQ7 [101]. Using three-dimensional mapping of their COQ7 cryo-EM structure, Manicki et al. suggested that the motif E X_6_ Y X_22_ E X_2_ H X_48_ E X_6_ Y X_28_ E X_2_ H is functionally important in forming these di-iron carboxylate ligands [52]. However, it is important to note that the structure determined for COQ7 did not possess metals, hence the residues that function to ligand the di-iron are surmised based on structural similarity to other di-iron carboxylate proteins [52]. SNVs at or near residues in the motif are predicted to disrupt the ability of COQ7 to ligand iron atoms. We encounter three SNVs at or near residues in the predicted di-iron-liganding motif. G59R and E60K occur at or adjacent to E60, which ligands iron 1 (Figure 27B). G59R was reported in Missense3D-DB and was characterized as altering a cavity by Missense3D. This is likely to reduce substrate access to the active site. E60K was reported in gnomAD and was characterized as switching a buried charge by Missense3D. It is important to note that Missense3D does not take into account the roles played by residues that act as metal ligands when making predictions about functionality.

The V141E variant occurs adjacent to E142, which is predicted to ligand iron 2 (Figure 27B). This variant was reported in the ClinVar database and is labeled as deleterious. Missense3D characterizes this variant as neutral. It is likely that V141E disrupts ligand formation with the iron.

#### 3.5.4. SNVs Predicted to Impact Redox Chemistry of COQ7

COQ7 utilizes NADH to reduce the iron atoms, which then activate O_2_ to perform the hydroxylation step of the DMQ substrate [101]. Several of the residues predicted to act as ligands for the two iron atoms (E60, H148, Y149) are thought to mediate a sequential electron-proton-electron relay [101], and a DMQ quinone may act as a conduit between NADH and the di-iron site [105]. Intriguingly, Manicki et al. observed that the NADH cofactor is localized adjacent to a water-filled channel formed by the triad [52]. The variants E60K and Y149C discussed previously (Figure 27) likely disrupt this redox chemistry.

### 3.6. COQ9

COQ9 is peripherally associated with the IMM and is shown to be essential for CoQ synthome formation and stabilization in both human and yeast cells [50,106]. COQ9 homologs play slightly different roles in different organisms. Coq9, the yeast COQ9 homolog, plays a supportive role in the hydroxylation steps mediated by Coq6 and Coq7 [13]. Certain *coq9* yeast mutants accumulate demethoxy-Q (DMQ), the penultimate intermediate of the CoQ biosynthetic pathway [107]. In humans, COQ9 plays a supportive role in the COQ7 hydroxylation of DMQ in addition to the stabilization of the CoQ synthome [25]. Patients with deficiencies in COQ9 have been shown to accumulate DMQ_10_, the same intermediate that accumulates in COQ7-deficient cells [108].

To date, pathogenic cases of genetic variants in the *COQ9* gene have been identified in seven patients across four families [29]. While the determination of pathogenic variants in *COQ9* is rare, mutations in the *COQ9* gene are associated with primary CoQ_10_ deficiency and are further associated with several disease states, predominantly encephalomyopathy and an autosomal-recessive neonatal-onset CoQ_10_ deficiency [29]. Low levels of CoQ_10_ in human cells resulting from COQ9 deficiency can be partially restored by treatment with 2,4-diHB or VA [109]. For 2,4-diHB, the addition of the hydroxyl group at the 2-carbon position allows it to bypass the COQ7 hydroxylation step in COQ7-deficient cells. VA was shown to increase the production of CoQ_10_ in COQ9-deficient cells [109]. Neither 2,4-diHB nor VA have been used as treatments in patients.

**Figure 28:**
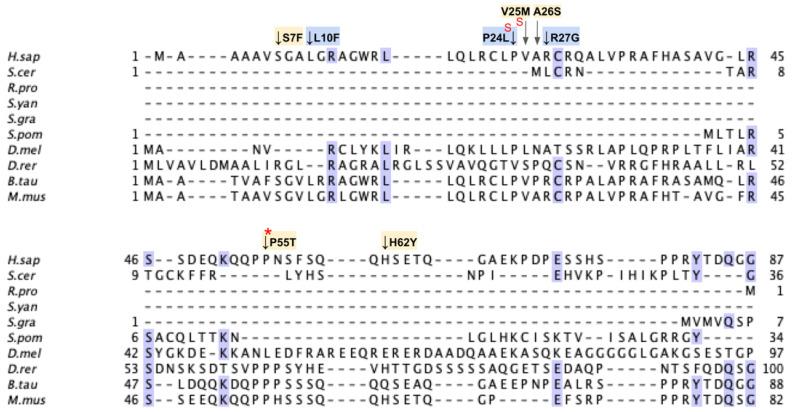

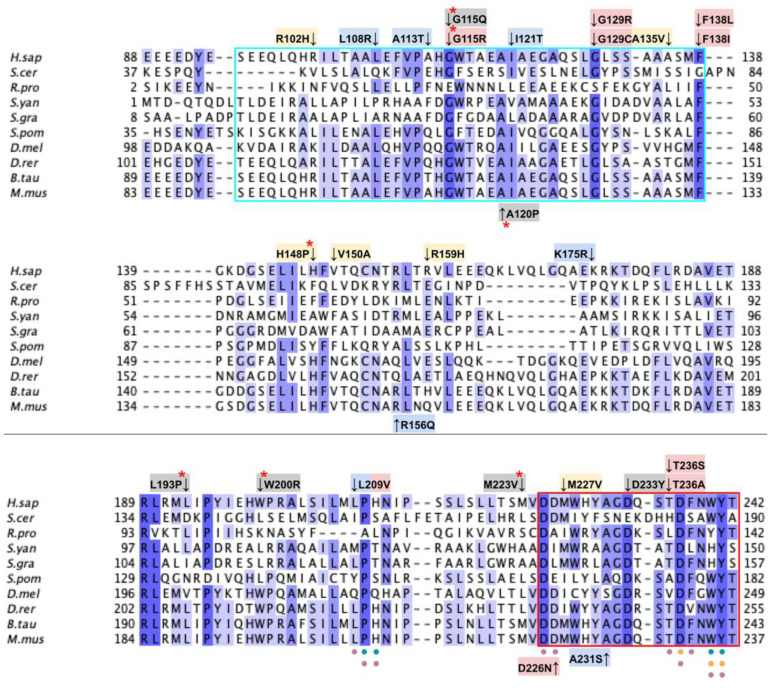

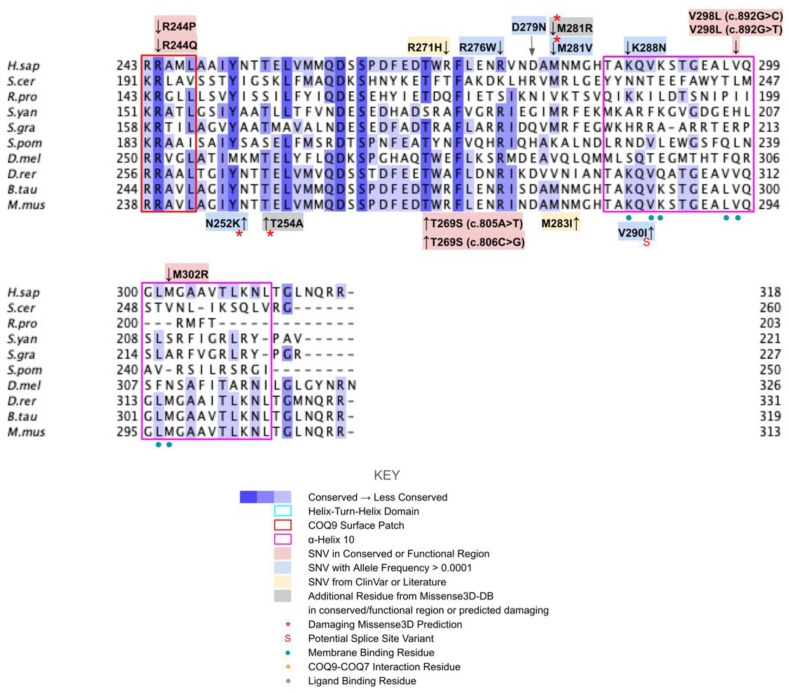
Labeled and annotated multiple sequence alignment of COQ9. Amino acid sequences of COQ7 were analyzed as described in Materials and Methods and include *Homo sapiens* (NCBI accession number NP_064708.1) and homologs in *Saccharomyces cerevisiae* (QHB10353.1), *Rickettsia prowazekii* (WP_004596292.1)*, Sphingomonas yanoikuyae* (EKU74250.1)*, Sphingopyxis granuli* (WP_082737038.1)*, Schizosaccharomyces pombe* (NP_594426.1), *Drosophila melanogaster* (NP_724594.1), *Danio rerio* (NP_001092216.1), *Bos taurus* (NP_001039767.1), and *Mus musculus* (NP_080728.1). See KEY for descriptions of the figure annotations. Residues in the HTH domain are boxed in cyan and COQ9 surface patch in red, data from [25]. COQ7 interaction residues are marked with orange dots, data from [52]. Ligand-binding residues are marked with purple dots, data from [50].

From gnomAD, ClinVar, and Missense3D-DB, we identified 52 missense SNVs of interest (Figure 28; Supplemental Table S1). Of these variants, 17 coincided with a highly conserved region or functional residue, which were determined from the multiple sequence alignment as well as structural studies on human COQ9 [50,52]. Two of the twelve variants classified as structurally damaging by Missense3D were contained in this group (Figure 28). From the ClinVar Database and literature, 11 missense variants were determined to be pathogenic or likely pathogenic. Two of these variants (P55T and H148P) were identified by Missense3D to be structurally damaging. There were 17 SNVs in gnomAD identified as frequent polymorphisms. Two of these, N252K and M281V (allele frequencies of 1.41 × 10^−4^ and 1.70 × 10^−4^, respectively), were identified as being structurally damaging by Missense3D.

**Figure 29:**
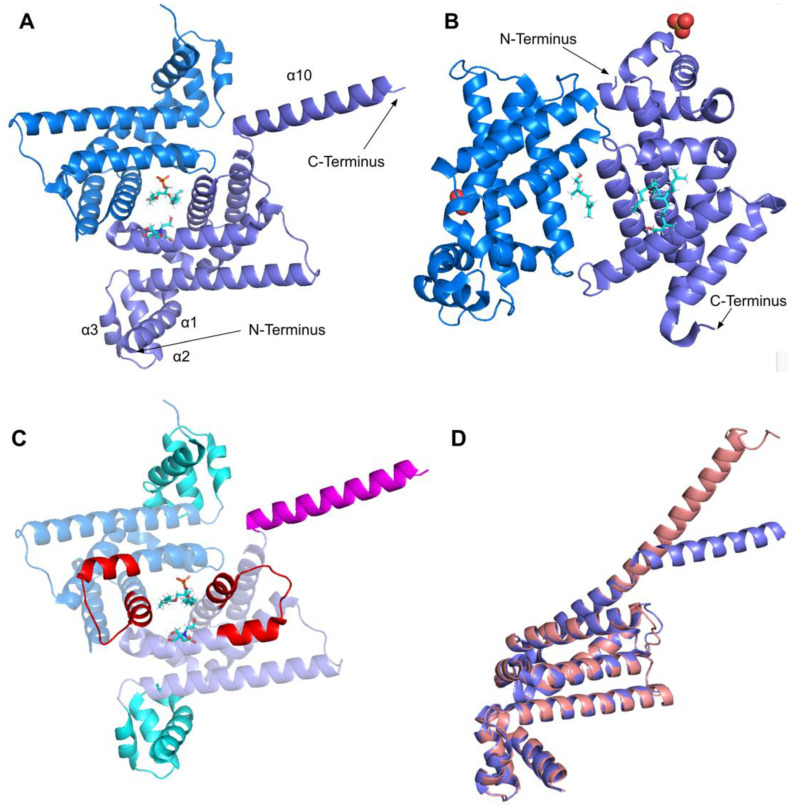
Comparison of existing structures of COQ9 and the AlphaFold model of COQ9 (**A**) PDB: 6AWL structure of COQ9^ΔN79^ with co-crystallized phosphatidylethanolamine and bis-tris shown in cyan, data from [50]. Chain A colored in purple, chain B colored in blue. (**B**) PDB: 6DEW structure of COQ9^ΔN79,ΔC36^ with co-crystallized geraniol, cis-trans-farnesol, trans-trans-farnesol, and cis-cis-farnesol shown in cyan and sulfate molecules shown as ball structures; Chain A colored in purple, chain B colored in blue, data from [50]. (**C**) Structural motifs shown on 6AWL structure. Colors match Figure 28. (**D**) Structural alignment of 6AWL chain A shown in purple with the AlphaFold model obtained from the AlphaFold Protein Structure Database [39,54], shown in pink.

The human COQ9 structure determined by X-ray diffraction at 2.00 Å resolution by Lohman et al. (PDB: 6AWL) was used as a model for visualizing the protein in PyMOL [50]. The COQ9 protein exists as a dimer with a hydrophobic cavity. Two co-crystallized molecules are visible in this structure: phosphatidylethanolamine and bis-tris (Figure 29A). A second COQ9 structure, determined by X-ray diffraction at 2.00 Å resolution by Lohman et al., PDB: 6DEW, co-crystallized with various isoprenes (Figure 29B) [50]. Additionally, Lohman et al. expressed COQ9^ΔN79^ (lacking the amino-terminal 79 residues) in *E. coli* and found six co-purified enriched lipids including CoQ_8_, menaquinone-8, and 2-octaprenyl phenol, which had the highest intensity signal of any lipid.

**Figure A4:**
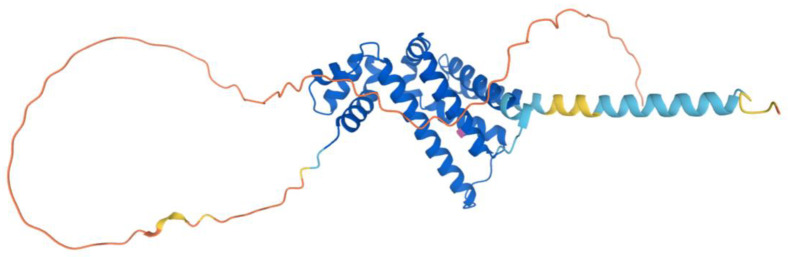
AlphaFold model of single chain of COQ9. Model color corresponds to the model confidence described in Figure 4. Figure was generated by the AlphaFold Protein Structure Database [39,54].

This indicates COQ9 as lipid-binding and its hydrophobic pocket as a potential binding site. Structural motifs noted in Figure 28 are designated on the 6AWL structure (Figure 29C). A model for human COQ9 was obtained using the AlphaFold Protein Structure Database (Figure A4) [39,54]. This model shows 79 residues at the N-terminus that the crystal structure for COQ9 (PDB: 6AWL) lacked (Figure 29D).

#### 3.6.1. SNVs in the Helix-Turn-Helix Domain

**Figure 30:**
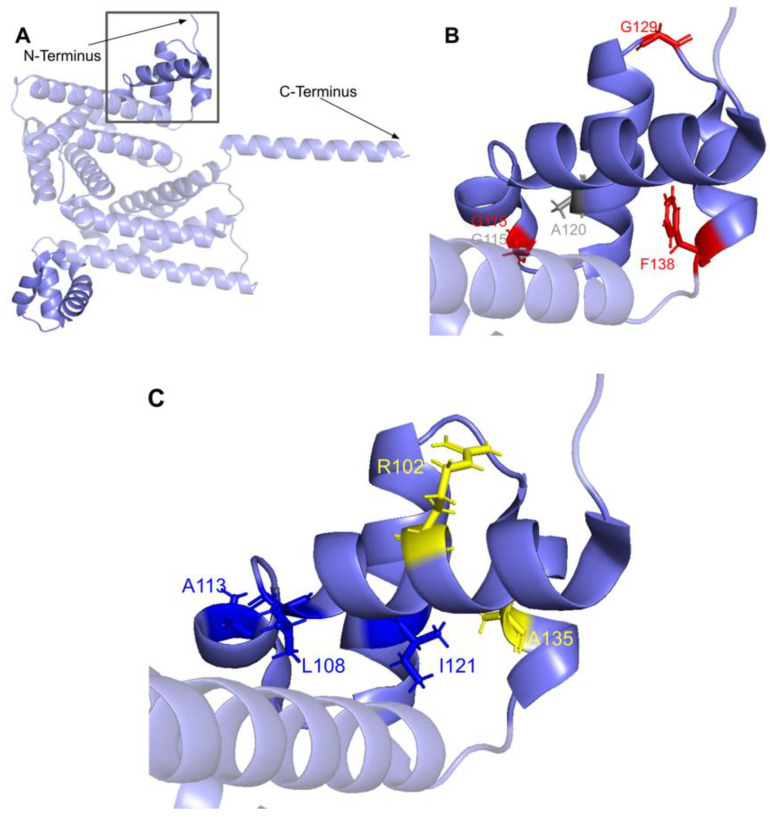
SNVs in the HTH-domain of COQ9. (**A**) N-terminal HTH-domains highlighted on the COQ9 dimer structure (PDB:6AWL), corresponding to residues S95 to F138 and D92 to F138, on chains A and B, respectively. (**B**) Locations of SNVs in conserved or functional regions are depicted. (**C**) Locations of ClinVar variants and frequent polymorphisms are depicted. Residues are colored according to their corresponding SNVs in Figure 28.

COQ9 has been determined to have structural homology to proteins in the TetR family of regulators (TFRs) [25]. TFR proteins are typically found in prokaryotes with small-molecule binding capability, allowing many in the family to regulate transcription [110]. All TFR proteins consist of an N-terminal helix-turn-helix (HTH)-domain DNA binding motif consisting of α-helices 1–3, or residues E91-F138 (Figure 30A) and a larger C-terminal ligand-binding motif consisting of α-helices 4–9 (Figure 29A). However, COQ9 is not predicted to bind DNA, making it an atypical TFR protein [25].

In the COQ9 HTH domain, we encounter twelve SNVs with relevance to this analysis. There are five SNVs reported in gnomAD occurring at highly conserved residues in this segment of amino acids: G115R, G129R, G129C, F138L, and F138I (Figure 30B). Of these five SNVs from gnomAD, only G115R was flagged by Missense3D as damaging. None of these variants are present as homozygotes in gnomAD, nor are they associated with a deficiency in CoQ_10_. Additionally, there are two SNVs reported in Missense3D-DB that are predicted to be structurally damaging by Missense3D: G115Q and A120P.

Three SNVs reported in gnomAD are identified as frequent polymorphisms: L108R, A113T, and I121T (Figure 30C). Only one variant, L108R, occurs at a highly conserved residue. According to the ClinVar database, one report states that L108R is likely benign whereas four others list an uncertain significance. Two of the four uncertain significance interpretations list primary CoQ_10_ deficiency as the associated condition. The L108R variant is of particular interest because of its frequency and possible pathogenicity; however, no homozygotes have been reported to date. A113T has been determined to be a likely benign SNV. I121T occurs at a semi-conserved residue, and there are conflicting reports ranging from being likely benign to having uncertain significance. All of these variants are characterized as neutral by Missense3D. Two additional SNVs are reported in ClinVar in the HTH-domain: R102H and A135V (Figure 30C). Both of these variants potentially lead to primary CoQ_10_ deficiency; however, the association must be investigated further in order to elucidate a certain link. Both SNVs are characterized as neutral by Missense3D.

#### 3.6.2. SNVs in the COQ9 Surface Patch

**Figure 31:**
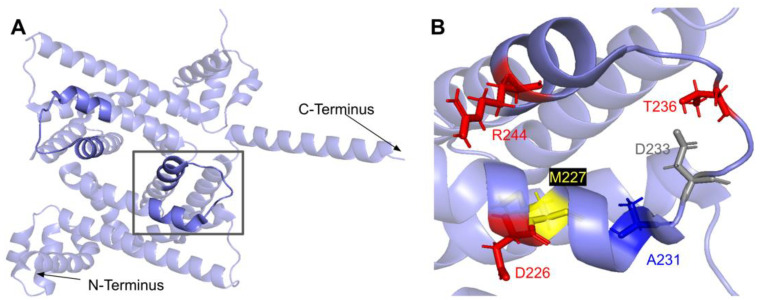
SNVs found in the COQ9 surface patch. (**A**) Surface patch highlighted on the COQ9 dimer (PDB:6AWL), corresponding to residues D225 to L247. (**B**) Locations of SNVs are depicted. Residues are colored according to their corresponding SNVs in Figure 28.

The second motif present in typical TFR proteins is a C-terminal ligand-binding domain [110]. The COQ9 surface patch is comprised of several highly conserved residues necessary for interaction with COQ7, ranging from residues D225 to L247 [25]. The surface patch resides near the lipid-binding pocket (Figure 31A). Mutagenesis of conserved residues in this region of COQ9 and co-purification with COQ7 has been shown to disrupt COQ9-COQ7 interaction, resulting in reduced production of CoQ and accumulation of the COQ7 substrate DMQ. Thus, we predict that mutations in highly conserved regions along the surface patch may result in either disruption of protein-protein interaction within the CoQ synthome or destabilization of the COQ9 protein itself. Variants in this region are not likely to disrupt protein stability, as these are solvent-exposed and will thus appear neutral to Missense3D.

In COQ9, the surface patch stretches from the end of α-helix 7 and the beginning of α-helix 8. In this domain, we encounter eight SNVs with relevance to this analysis. There are five SNVs reported in gnomAD occurring at highly conserved or functional residues in this segment of amino acids (Figure 31B). None of these mutations are predicted to be structurally damaging by Missense3D. None of these mutations has a clinical association with CoQ_10_ deficiency.

There is one additional SNV reported in Missense3D-DB occurring at a highly conserved residue: D233Y. This mutation is predicted to be structurally neutral by Missense3D. D233Y does not have a clinical association with CoQ_10_ deficiency. Another SNV reported in gnomAD is identified as a frequent polymorphism, A231S. This variant does not occur at a highly conserved residue. It is predicted to be neutral by Missense3D and has been labeled as likely benign by ClinVar. There is one SNV reported in ClinVar in the surface patch region, M227V (Figure 31B). This variant is associated with primary CoQ_10_ deficiency; however, it has been labeled as having unknown significance in ClinVar. M227V is characterized as neutral by Missense3D.

#### 3.6.3. SNVs in α-Helix 10

α-helix 10 (α10), a C-terminal amphipathic helix not present in typical TFR proteins, has been found to have importance in membrane binding [50]. Lohman et al. found that COQ9 binds the matrix side of the IMM in a series of five steps. In the second step, the hydrophobic residues along α10 associate with the membrane phospholipids. Mutating these hydrophobic residues to Ser significantly reduced liposome association. We predict that, despite low evolutionary conservation of residues along α10, mutations occurring at residues implicated in membrane binding may result in reduced membrane association and lower levels of synthome formation, thus resulting in lowered CoQ production.

**Figure 32:**
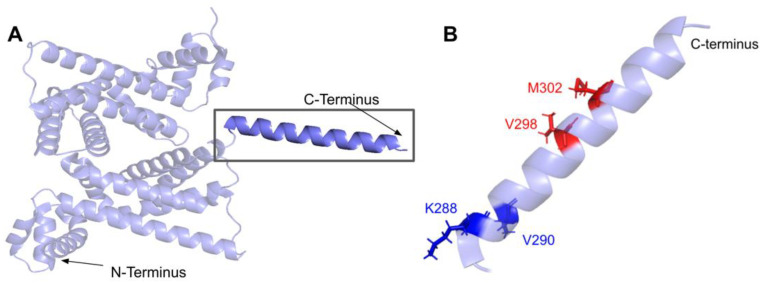
SNVs found in α10 of COQ9. (**A**) α10 highlighted on COQ9 dimer structure (PDB: 6AWL), corresponding to residues T286 to L311. (**B**) Locations of SNVs are depicted. Residues are colored according to their corresponding SNVs in Figure 28.

α10 occurs at the C-terminus of COQ9 from T286 to R311 (Figure 32A). In this domain, we encounter five SNVs with relevance to this analysis. Three SNVs reported in gnomAD occur at residues implicated in membrane binding: V298L (c.892G>C and c.892G>T) and M302R (Figure 32B). Both variants are characterized as neutral by Missense3D and neither are clinically associated with primary CoQ_10_ deficiency. For V298L, the SNV mutates a hydrophobic residue to another hydrophobic residue, minimizing the impact of the change. For M302R, the SNV mutates a hydrophobic residue to a positively charged residue, which may potentially have a large impact on membrane binding ability. There are two SNVs reported in gnomAD that were identified as frequent polymorphisms: K288N and V290I. Both variants are characterized as neutral by Missense3D and K288N is labeled as benign by ClinVar. V290I did not appear in ClinVar.

## 4. Discussion

Herein, we analyzed the functional and structural impacts of missense SNVs in *COQ3–COQ7* and *COQ9*. From a large pool of genome and exome sequencing data (gnomAD and Missense3D-DB), as well as clinically documented variants (NCBI ClinVar and literature), 429 variants were selected; these consisted of SNVs in conserved and functional regions, with potential clinical significance or with high allele frequency. Of these, 115 SNVs were classified as potentially structurally damaging by the Missense3D server. AlphaFold models were used for classification wherever crystal structures were not available. As expected, we found frequent polymorphisms to be less deleterious than those found in highly conserved or functional residues. The mutation classifiers used, Missense3D, SIFT, and PolyPhen-2, often had disagreements with their respective assessments. Some of this disagreement can be attributed to the assessment criteria used by each classifier. Missense3D is more stringent in its structural consequence criteria whereas the SIFT and PolyPhen-2 algorithms are less conservative when classifying a mutation as deleterious. Nevertheless, when the data obtained across classifiers are compared in the context of function, one can make an educated prediction based on the considerations described here.

It is imperative to note that predicted models may not accurately reflect the true physiological structures. The dynamic residue-residue interactions between polypeptides may result in different local conformations that are not represented in the individual structural models, including disordered regions. A further consequence of this limitation is that mutation classifiers such as the ones used in this study cannot predict the extent to which the activity of an enzyme is affected. Additionally, mutation classifiers may underestimate the deleteriousness of certain mutations, such as point mutations within binding pockets deemed “benign” that may impair essential cofactor or substrate binding, polymorphisms within the mitochondrial targeting sequence that may affect localization, or surface mutations that may alter protein-protein interaction interfaces. In the case of Missense3D, it is currently unable to model mutations at a protein-protein or protein-ligand interaction interface, as additional polypeptide chains or ligands in the provided structure are removed during analysis. One example of a mutation that was overlooked by Missense3D is the D208H mutation in COQ6, which occurs at a putative FAD-binding residue. This mutation has been associated with schwannomatosis in a family of patients [96]. While this linkage has been contested, it has been shown that human COQ6 harboring this mutation is unable to rescue *coq6* null yeast, suggesting a loss of function. However, Missense 3D had classified the mutation as neutral.

Differential gene expression in distinct tissues may also account for pathogenic effects that cannot be characterized solely by predicted structures. For example, mutations associated with the atypical kinase COQ8B present a perplexing case where the pathogenic phenotypes appear to be kidney-specific [29]. While the methods described in this study can identify potentially pathogenic SNVs, they cannot predict the extent to which a structurally destabilizing mutation decreases protein levels in a given tissue, nor whether a resulting decrease is sufficient to affect tissue function. It is also worth noting that distinct cell types may also be differentially impacted by CoQ deficiency due to particular energy demands or exposure to oxidative damage.

Despite these limitations, multiple sequence alignments and structural models have revealed highly conserved and functionally relevant residues, which allowed us to narrow our search in identifying variants with potential clinical significance. The pipeline used in this work may also serve as a scaffold for future SNV studies. Multiple sequence alignments can help identify highly conserved regions of unknown function such as post-translational modification sites. Meanwhile, structural models can be used in computational studies to predict protein-protein interaction interfaces as well as the effect of SNVs on ligand binding and catalysis. All of these studies are invaluable in elucidating the regulatory mechanisms of CoQ biosynthesis. Furthermore, our pipeline provides a structural basis for analyzing existing pathogenic mutations. For example, Ling et al. identified a patient with a homozygous mutation G124S in COQ4 inherited from heterozygous parents. Skin fibroblasts isolated from the patient exhibited low CoQ_10_ content [111]. A separate study found another patient homozygous for the G124S allele with poor cardiac contractility and developmental delays [71]. In silico analyses from Missense3D, SIFT, and Polyphen-2 all correctly predicted the deleterious nature of this mutation. In particular, Missense3D classified the mutation as damaging due to the replacement of a buried Gly, suggesting deleterious structural consequence. Additionally, this pipeline can be combined with other publicly available high-throughput data, such as gene expression profiles of different human tissues, to yield further insight into the phenotype variability in primary CoQ deficiencies.

Primary CoQ_10_ deficiency is often caused by autosomal recessive mutations. Compounded with the limited accessibility to genome sequencing, it is likely that a significant portion of pathogenic variants has not been identified. Furthermore, early intervention of primary CoQ_10_ deficiency has shown to be effective [32], illuminating the importance of detecting such mutations early. As the cost of genetic sequencing declines over time, we expect more variants to be identified in the future.

To aid the prompt diagnosis of pathogenic variants causing primary CoQ_10_ deficiency, further in vitro and in vivo biochemical characterizations in model organisms such as *S. cerevisiae* are required to validate the SNV classifications made in this study. Regarding COQ3 and COQ5, there has been little to no clinical information on *COQ3*- and *COQ5*-associated primary CoQ_10_ deficiency. This may appear to suggest that COQ3 and COQ5 mutations are not pathogenic. However, we speculate that many structurally damaging variants may be embryonic lethal, resulting in an apparent lack of known pathogenic variants. Conversely, mutations in the COQ8A and COQ8B polypeptides are reported to have several pathogenic effects [29], likely due to the aforementioned dynamic interactions and/or redundancy of function. For this reason, the clinical effects of mutations in the COQ8A and COQ8B polypeptides would require a more elaborate structural analysis and stringent biochemical characterization to fully understand the consequences of such SNVs.

Current treatment for CoQ deficiency relies on oral supplementation with CoQ_10_. However, this method is only partially successful due to poor uptake of this extraordinarily hydrophobic lipid molecule. It will be important to identify the gene products responsible for the cellular trafficking of both exogenous and endogenous CoQ and its delivery to the mitochondria [112,113]. A potential alternative is a “bypass” treatment, in which a CoQ precursor can be administered to circumvent any faulty enzymatic steps in the biosynthetic pathway, as demonstrated previously in yeast [90], mice [114], and human fibroblasts [115]. Unfortunately, the success of this potential avenue is contingent upon the nature of the mutation. The COQ polypeptide harboring the SNV must be able to retain a structurally stable CoQ complex. Given the extensive interactions between the COQ polypeptides, membrane association, and binding of CoQ and CoQ-biosynthetic intermediates in the high molecular mass CoQ synthome, it seems likely that many surface residue SNVs may impact these interactions.

Similar to the bypass approach, another option for treating CoQ deficiency is supplementation with MitoQ. MitoQ is composed of the lipid molecule conjugated to a triphenylphosphonium cation, which enables the antioxidant to be targeted to the mitochondria [116]. Recently, clinical studies involving the supplementation of this CoQ derivative have been shown to attenuate mitochondrial DNA damage in skeletal muscle [117,118], although the direct role of MitoQ in this protective effect remains unclear. Given that MitoQ does not support the respiratory electron transport function of CoQ, it is unlikely on its own to rescue primary CoQ_10_ deficiency [119].

A recent innovative method to serve as a potential therapy for mitochondria-related diseases is mitochondria transplantation. The fusion and fission of mitochondria within the cell are essential for the maintenance of healthy mitochondrial functions [120], and such dynamics are an attractive process to exploit for therapeutic applications. Recent studies explored the potential for mitochondrial transplants as a targeted therapy for repairing defunct mitochondria in different cells and, in a larger application, tissue revitalization [121,122]. Similarly, given the association of mitochondrial dysfunction with disease, another avenue for therapeutics involves the design of drugs that induce mitochondrial biogenesis, thereby promoting the turnover of defunct mitochondria and subsequently the emergence of healthy mitochondria. Several naturally occurring polyphenols have been identified as mitochondrial biogenesis inducers, such as resveratrol and phytoestrogens [123]. However, similar to oral CoQ_10_ supplements, the therapeutic advantage of these inducers is limited by poor absorption. To target mitochondrial biogenesis from a transcriptional approach, a number of nuclear transcription factors have also been implicated in promoting mitochondrial biogenesis, which would help coordinate the expression of both mitochondrial-encoded genes as well as those that are nuclear-encoded, such as our *COQ* genes [123,124]. Unfortunately, the response resulting from this approach would be nonspecific, as targeting the induction of transcription factors would activate several other pathways, potentially leading to more severe side effects. Currently, targeting mitochondrial disease through the induction of mitochondrial biogenesis remains unsuccessful. While these approaches may have promise for treating mitochondrial dysfunctions, primary CoQ_10_ deficiencies result from mutations in the nuclear-encoded *COQ* genes, making this method suitable for only certain types of secondary CoQ_10_ deficiencies.

In summary, this work provides a thorough structural and functional analysis of clinically relevant SNVs in several of the *COQ* genes. We have identified 115 SNVs that are likely pathogenic due to structural perturbations, using multiple sequence alignments, mutation classifiers, and predicted or solved protein structures. This analysis highlights the intricate interactions in local regions of a given protein, as well as the large-scale dynamic interactions between the individual constituents of protein complexes. Together, this serves as a scaffold for future studies that seek to characterize the biochemical consequences of SNVs that result in pathogenic effects caused by primary CoQ_10_ deficiencies. Finally, the approach employed takes advantage of recent advances in protein tertiary structure prediction and should be applicable to any human gene with identified homologs and reported SNVs.

## Data Availability

All models and crystal structures used in Missense3D analysis were obtained from the AlphaFold Protein Structure database (https://alphafold.ebi.ac.uk/ accessed on 14 October 2022) and the Protein Data Bank (https://www.rcsb.org/ accessed on 14 October 2022), respectively. Accession numbers of these structures can be found in the Materials & Methods. Accession numbers of the protein sequences analyzed in this study can be found in the captions of Figures showing the respective multiple sequence alignments. All SNVs analyzed in this study were obtained from gnomAD (https://gnomad.broadinstitute.org/ accessed on 30 March 2022), ClinVar (https://www.ncbi.nlm.nih.gov/clinvar/ accessed on 30 March 2022), and Missense3D-DB (http://missense3d.bc.ic.ac.uk:8080/ accessed on 30 March 2022). A full list can be found in Table S1.

## Supplementary Materials

The following supporting information can be downloaded at: https://www.mdpi.com/article/10.3390/antiox11122308/s1, Table S1: Comprehensive list of analyzed variants. References [71,72,74,90,91,92,93,96,99,100,103,111,115,125,126,127,128,129,130,131,132,133,134,135,136,137,138,139,140,141,142,143,144,145,146,147,148] were cited in supplementary.

## Abbreviations

AdoHcy, S-adenosyl-L-homocysteine; AdoMet, *S*-adenosyl-L-methionine; apo, apoenzyme; *APTX*, gene encoding aprataxin; Arh1, yeast ferredoxin reductase; *BRAF*, gene encoding B-Raf; CoQ, coenzyme Q or ubiquinone; CoQH_2_, reduced CoQH_2_ hydroquinone or ubiquinol; CoQn, *n* = number of isoprene units in polyprenyl tail; DDMQH_2_, 2-methoxy-6-polyprenyl-1,4-benzohydroquinone; DHP, 4,5-dihydroxy-3-polyprenylphenol; DHPB, 4,5-dihydroxy-3-polyprenylbenzoic acid; 2,4,-diHB, 2,4-dihydroxybenzoic acid; 3,4-diHB, 3,4-dihydroxybenzoic acid; DMAPP, dimethylallyl pyrophosphate; DMeQH_2_, 3-methyl-6-methoxy-2-polyprenyl-1,4,5-benzenetriol; DMQH_2_, 2-methoxy-5-methyl-6-polyprenyl-1,4-benzohydroquinone; *ETFDH*, gene encoding mitochondrial electron transfer flavoprotein-ubiquinone oxidoreductase; FDX1, human ferredoxin 1; FDX2, human ferredoxin 2; FDXR, human ferredoxin reductase; HAB, 3-hexaprenyl-4-aminobenzoic acid; 4-HB, 4-hydroxybenzoic acid; HHB, 3-hexaprenyl-4-hydroxybenzoic acid; HMPB, 4-hydroxy-5-methoxy-3-polyprenylbenzoic acid; 4-HP, 4-hydroxy-3-polyprenylphenol; 4-HP_6_, 3-hexaprenyl-4-hydroxyphenol; HPB, 3-polyprenyl-4-hydroxybenzoic acid; IMM, inner mitochondrial membrane; IPP, isopentenyl pyrophosphate; *MUT*, gene encoding methylmalonyl-CoA mutase; pABA, para-hydroxybenzoic acid; PDB, protein data bank; PDSS1, decaprenyl diphosphate synthase subunit 1; PDSS2, decaprenyl diphosphate subunit 2; SNV, single nucleotide variant; SNHL, sensorineural hearing loss; SRNS, steroid-resistant nephrotic syndrome; TFRs, TetR family of regulators; UbiG, *E. coli* ubiquinone biosynthesis *O*-methyltransferase; VA, vanillic acid; Yah1, yeast ferredoxin.
